# Spatiotemporal view of malignant histogenesis and macroevolution via formation of polyploid giant cancer cells

**DOI:** 10.1038/s41388-022-02588-0

**Published:** 2023-01-03

**Authors:** Xiaoran Li, Yanping Zhong, Xudong Zhang, Anil K. Sood, Jinsong Liu

**Affiliations:** 1 Department of Anatomical Pathology, Division of Pathology and Laboratory Medicine, Houston, TX USA; 2 Department of Pathology, The First Hospital of Jilin University, Changchun, 130021 Jilin China; 3 Department of Gynecologic Oncology and Reproductive Medicine, The University of Texas MD Anderson Cancer Center, 1515 Holcombe Boulevard, Houston, TX 77030-4095 USA

**Keywords:** Tumour heterogeneity, Cancer microenvironment, Mitosis, Cellular imaging

## Abstract

To understand how malignant tumors develop, we tracked cell membrane, nuclear membrane, spindle, and cell cycle dynamics in polyploid giant cancer cells (PGCCs) during the formation of high-grade serous carcinoma organoids using long-term time-lapse imaging. Single cells underwent traditional mitosis to generate tissue with uniform nuclear size, while others formed PGCCs via asymmetric mitosis, endoreplication, multipolar endomitosis, nuclear fusion, and karyokinesis without cytokinesis. PGCCs underwent restitution multipolar endomitosis, nuclear fragmentation, and micronuclei formation to increase nuclear contents and heterogeneity. At the cellular level, the development of PGCCs was associated with forming transient intracellular cells, termed fecundity cells. The fecundity cells can be decellularized to facilitate nuclear fusion and synchronized with other nuclei for subsequent nuclear replication. PGCCs can undergo several rounds of entosis to form complex tissue structures, termed fecundity structures. The formation of PGCCs via multiple modes of nuclear replication in the absence of cytokinesis leads to an increase in the nuclear-to-cytoplasmic (N/C) ratio and intracellular cell reproduction, which is remarkably similar to the mode of nuclear division during pre-embryogenesis. Our data support that PGCCs may represent a central regulator in malignant histogenesis, intratumoral heterogeneity, immune escape, and macroevolution via the de-repression of suppressed pre-embryogenic program in somatic cells.

## Introduction

Pathologists have long observed that cancer cells exhibit abnormal nuclear morphology with increased cell size and genome copy number, commonly referred to as nuclear atypia [1, 2]. The abnormal nuclear features include nuclear pleomorphism, high nuclear to cytoplasmic (N/C) ratio, polyploid mitosis, and mononucleated or multinucleated giant cells, i.e., polyploid giant cancer cells (PGCCs). These nuclear abnormalities represent the most critical histologic criterion for making a cancer diagnosis [3]. Abnormal nuclear morphology was shown to correlate with high-level malignancy and poor prognosis in cancer patients [4–8].

Giant cell size in cancer is commonly associated with increased genomic contents or polyploidy via whole genomic duplication or multiplication. It is well known that polyploidy plays a crucial role in tumorigenesis and development [9–12]. Polyploid genomes exist in 37 to 50% of high-grade cancers [13–15]. The cancer genomic atlas projects show cancers have multiple structural defects, including polyploid genome, chromothripsis [16, 17], and extrachromosomal DNA [18, 19]. Increased genomic content invariably leads to the formation of PGCCs. However, the role of PGCCs in the development of malignant tumor histogenesis and evolution is largely unknown.

For a long time, PGCCs were considered non-dividing senescent cells because of their inability to execute mitosis. However, we and others have demonstrated that PGCCs are viable and capable of generating mitotically active daughter cells via amitotic mechanisms [20–23]. PGCCs were shown to give rise to viable daughter cells following stresses induced by irradiation [21, 24, 25], chemotherapy [22, 26–30], a hypoxia-mimetic agent cobalt chloride (CoCl_2_) [31], cellular or viral oncogene-induced senescence [32], and gene mutation in leukemia cells [33]. PGCCs have also been shown to express embryonic stemness markers [27, 34]. Single PGCCs were shown to be capable of initiating tumor growth and metastasis [27, 31, 35] and recapitulating properties of early embryogenesis [27, 31, 36, 37].

Despite our current understanding of cancer polyploidy, the role of PGCCs in malignant tumor development in a 3-dimensional (3D) tissue microenvironment has not been studied. To address this knowledge gap, we applied long-term time-lapse live-cell imaging to track the cellular and nuclear dynamics of PGCCs during the development and evolution of patient-derived high-grade serous carcinoma (HGSC) organoids.

## Results

### Two types of organoids without or with fecundity cells

**Fig. 1:**
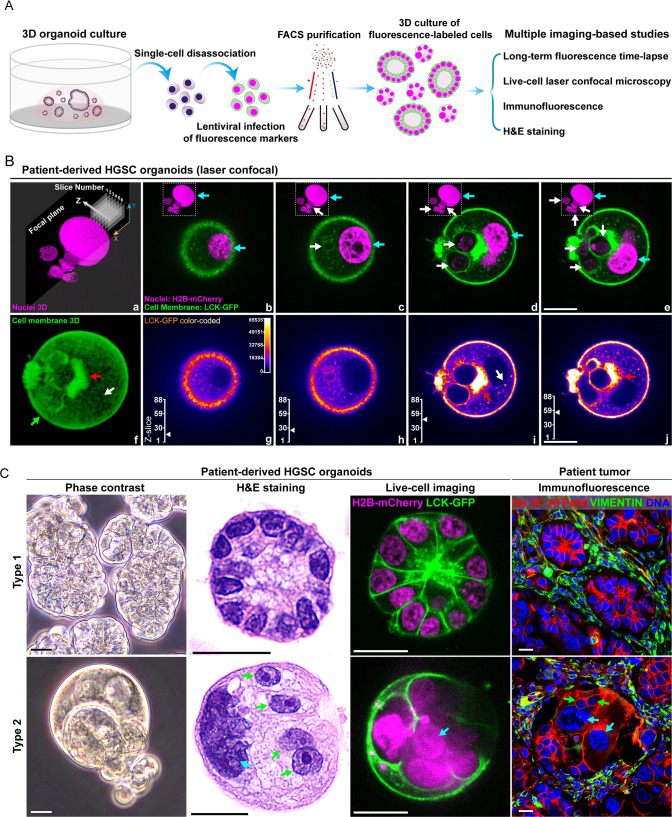
Histologic characteristics of type 1 and type 2 organoids. **A** The workflow for labeling patient-derived HGSC cells with fluorescence markers, cell purification, organoid culture, and imaging-based studies. FACS, fluorescence-activated cell sorting, H&E, hematoxylin-eosin. **B** Representative type 2 MDA-HGSC-2414 HGSC organoid with fecundity cells captured by laser confocal microscopy with Z-axis scanning. a Reconstructed 3D image of nuclei (Z-scanning). b–e Merged fluorescence images of the cell membrane (green) and nuclei (magenta) from four Z-slices. Cyan arrows: PGCC nuclei; white arrows: fecundity cells. f The reconstructed 3D image demonstrates the overall cell membrane distribution and structures. Pixels were adjusted to 70% transparency to show the vesicle-rich region in the center of the organoid. g–j Color-coded LCK-GFP images. The images demonstrate the complex subcellular structures associated with membrane-rich vesicles and cell membrane borders of the fecundity cells. The Z-slice scales mark the position of the current image. For panels f–j: green arrow: cell membrane; red arrows: large membrane aggregate adjacent to fecundity cells; white arrows: solitary membrane aggregate in the cytoplasm. Bars equal 20 μm. **C** Key morphological characteristics of type 1 and type 2 organoids and the corresponding structures in patient tumors. From right to left: Live-cell imaging, phase contrast, fixed organoids, H&E-stained, bright field; Live-cell imaging, fluorescence laser confocal. LCK-GFP: green; H2B-mCherry: magenta; Fixed patient tumor tissue, wide-field epifluorescence. Immunofluorescence staining includes Na + /K + ATPase (epithelial cell membrane, red), Vimentin (mesenchymal cells, green), and DAPI (DNA, blue). Cyan arrow: giant nucleus; green arrows: fecundity cells. Bars equal 20 μm.

First, we established multiple HGSC organoid lines from patient-derived xenografts and primary patient tumors. Results for one organoid line, MDA-HGSC-2414, which recapitulated the morphologic heterogeneity of HGSCs, will be used for the following studies. The general workflow is shown in Fig. 1A.

The HGSC organoids are highly heterogeneous, and some display complex structures with PGCCs as their main frameworks. Laser confocal microscopy of a representative organoid with such complex structures revealed that these organoids were made up of numerous intracellular cells (Fig. 1B, panels a–e, white arrows) inside a host PGCC (Fig. 1B, panels b–e, cyan arrows, and Supplementary SV01-SECTION 1). As indicated by intense GFP fluorescence, membrane-associated aggregates were often observed in this type of organoid (Fig. 1B, panels f–j, red and white arrows, and Supplementary SV01-SECTION 2).

We termed these membrane-wrapped intracellular cells *fecundity cells* as *fecundity* relates to fruitfulness, pregnancy, or fertility.

According to the absence or presence of fecundity cells, the heterogeneous HGSC organoids could be divided into two broad subtypes. Type 1 organoids (Fig. 1C, top panels) accounted for ~20% of the total, which developed relatively uniform nuclei surrounded by the cell membrane, referred to as cellularized nuclei, and the cells formed glandular-like tissue structures. Type 2 organoids (Fig. 1C, bottom panels) accounted for ~80% of the organoids, which had variable numbers of giant nuclei (cyan arrows) with multiple fecundity cells (green arrows). This organoid line provides an ideal model for studying the development of cancer heterogeneity.

### Nuclear developmental pathway for two types of organoids

Next, we tracked the development of both organoid types from dissociated single cells expressing plasma membrane and nuclear fluorescence markers.

**Fig. 2:**
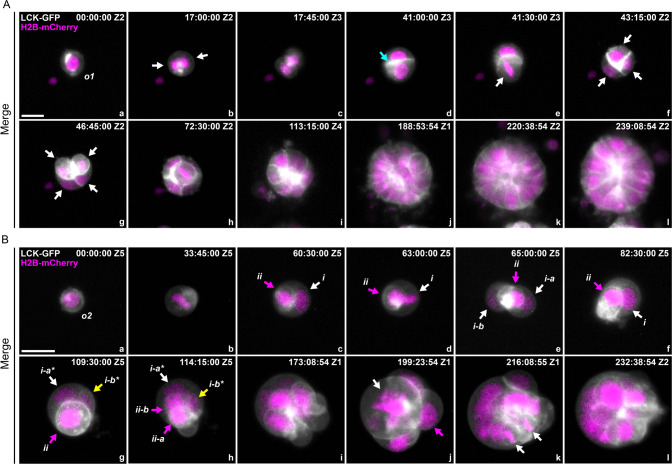
Development pathway of type 1 and type 2 organoids. Spatiotemporal view of development from a single cell to a multicellular organoid. Gray: cell membrane, LCK-GFP. Magenta: nuclei, H2B-mCherry. **A** The development of a type 1 organoid is dominated by canonical mitosis without forming any prominent fecundity cell structure. The sphere exhibits typical glandular features with relatively uniform cells. The original single cell is labeled o1. White arrows: original cell and its progeny cells; cyan arrow: cell-cell boundary. **B** The development of a type 2 organoid involves generating fecundity cells and forming complex tissue structures with pleomorphic nuclear morphology. The original single cell is labeled o2. The roman letters i and ii denote the daughter cells. The suffixes -a and -b indicate their generation; the suffixes -a* and -b* indicate the two daughter cells from the second round of mitosis. c–f White arrows: host PGCC, magenta arrows: fecundity cells. g, h White arrows: host PGCC; Magenta arrows: fecundity cell at metaphase and its daughter cells.; yellow arrows: a new fecundity cell (cell i-b*) generated from mitosis of cell-i. j White arrow: giant chromosome assembly at metaphase in the host PGCC; magenta arrow: released fecundity cell. k White arrows: bipolar mitotic figure from a daughter cell. The time format is hours: minutes: seconds. Bars equal 20 μm.

During the development of a representative type 1 organoid, the initial single cell (marked as o1, Fig. 2A, panel a) generated two daughter cells after the first mitosis (Fig. 2A, panel b, white arrows). The cell membrane boundary was observed in the center of this dividing sphere before it separated into two individual cells (Fig. 2A, panels c, d, cyan arrow). With time, the daughter cells underwent additional mitosis (Fig. 2A, panels e–g) and yielded four progeny cells (white arrows), which continued dividing and finally generated a larger glandular blastula-like tissue structure (Fig. 2A, panels h–l, and Supplementary SV02-SECTION 1). The corresponding fluorescence images of H2B-mCherry and LCK-GFP are shown in Supplementary Fig. S1A.

For the development of type 2 organoids, following the first mitotic division, the subsequent nuclear replication of the initial single cell could occur either without or with intra-cellularization. Representative examples of both forms of nuclear replication are shown in Fig. 2B. The initial single cell marked o2 (Fig. 2B, panel a) was similar in size to the single cell o1. Approximately 33.45 h after imaging began, the cell o2 entered metaphase, as indicated by condensed chromosomes aligned on the equatorial plate (Fig. 2B, panel b). At the end of this mitosis, two daughter nuclei (i and ii) were formed. Then, these two cells displayed different developmental processes.

Nucleus i increased its size via endoreplication (Fig. 2B, panel c, white arrow), then underwent mitosis at 63:00:00 (Fig. 2B, panel d, white arrow) and generated two daughter nuclei (i-a and i-b) (Fig. 2B, panel e, white arrows). However, the two nuclei failed to form two separated cells but fused into a polyploid nucleus (Fig. 2B, panel f, white arrow). The giant nucleus underwent asymmetric mitosis and generated two unequal-sized nuclei (i-a* and i-b*, white and yellow arrows, respectively), and the smaller nucleus (i-b*) was wrapped by the cell membrane and formed a new fecundity cell (Fig. 2B, panel g, yellow arrow).

Nucleus ii completed cellularization and formed the first fecundity cell (Fig. 2B, panels c, d). Notably, before the first mitosis of cell o2, the cell membrane displayed a “polarized” pattern, evidenced by the uneven distribution of membrane-associated fluorescence (Supplementary Fig. S1B, cyan arrows). This “polarized” region on the plasma membrane established a pocket-like membrane structure before the cell o2 entered metaphase (Fig. 2B, panel b). With the progression of mitosis, the “polarized” cell membrane pocket captured the daughter nucleus ii, thus forming the first fecundity cell (Fig. 2B, panel c, magenta arrow).

Fecundity cell ii underwent mitosis (d-e) and nuclear fusion (f). Then, the fused nucleus underwent new around mitosis (Fig. 2B, panel g) and yielded two daughter nuclei (Fig. 2B, panel h, ii-a and ii-b, magenta arrows), while cell ii-b also became a fecundity cell (Supplementary SV02-SECTION 2). Over five days, the fecundity cells underwent additional mitosis and generated new fecundity cells that formed a complex tissue structure (Fig. 2B, panels i–l, white arrows). This complex tissue structure formed by host PGCCs with intracellular fecundity cells will be termed fecundity structure. The corresponding color-coded LCK-GFP and H2B-mCherry fluorescence images are shown in Supplementary Fig. S1A and B.

The growth kinetics of the two types of organoids is shown in Supplementary Fig. S2. Quantitative data indicated that this typical type 1 organoid was faster in increasing its size but slower in accumulating DNA content. In contrast, the DNA synthesis was faster in the type 2 organoid (Supplementary Fig. S2, panels A-B). Consequently, type 2 organoids often displayed an increased N/C ratio compared to type 1 (Supplementary Fig. S2, panels C-D). Notably, with the maturity of type 2 organoids, the fecundity cells often become highly proliferative. A representative example is shown in Supplementary Fig. S3. The fecundity cells (00:00:00) rapidly migrated out from the host PGCC (33:00:00), exhibited robust mitotic activities, and formed a large cell colony (72:00:00).

### Nuclear augmentation via multipolar endomitosis or restitution multipolar endomitosis

Endomitosis refers to cells entering mitosis but not undergoing the steps to complete mitosis, including spindle assembly and segregation of sister chromatids within an intact nuclear envelope (classic endomitosis) or extended endomitosis including variant cell cycles in which aspects of mitosis occur, such as nuclear envelope breakdown, anaphase, and nuclear division, but without cytokinesis [38]. Multipolar endomitosis (MEM) refers to genome multiplication followed by complete separation of individual nuclei; Restitution multipolar endomitosis (RMEM) refers to the condensed chromosomes failing to separate at any stage of karyokinesis after initiation of mitosis, resulting in high pleomorphic nuclei with extensive nuclear fragmentation [39].

**Fig. 3:**
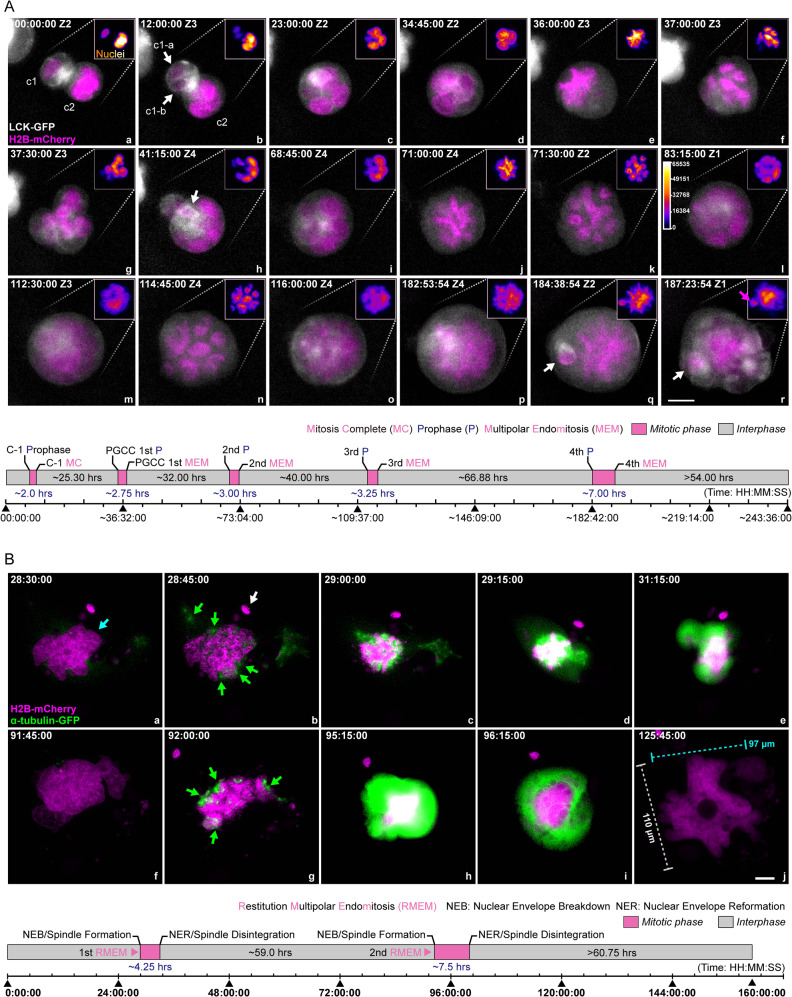
Multipolar endomitosis (MEM) and restitution multipolar endomitosis (RMEM). **A** A representative MEM event with partial cellularization. Following entosis, the two nuclei synchronized in cell cycles. The new PGCC underwent multiple endomitotic events followed by karyokinesis and internal cellularization to generate fecundity cells. Gray: cell membrane, LCK-GFP. Magenta: nuclei, H2B-mCherry. The two initially adherent cells are labeled as c1 (smaller cell) and c2 (host PGCC), and the progeny daughter cells of cell c1 are labeled as c1-a and c1-b (white arrows in panel b). The white arrow in panel h, fecundity cell generated during the first round of MEM; white arrows in panels q and r, fecundity cell generated during the fourth round of MEM; magenta arrow in panel r, chromatin aggregate encapsulated by a plasma membrane pocket. (Bottom panel) Schematic illustration of cell proliferation kinetics. The mitotic phase’s length was defined by the time interval between the start of prophase and the next nuclei re-formation. The time format is hours: minutes: seconds. Z with numbers indicates the z-slice position of the current image. Bar equals 20 μm. **B** A representative RMEM event was observed under a 2D condition. The spindle assembly indicates restitution mitosis (showing intensive green fluorescence) without complete separation of the condensed chromosomes. a–j Magenta: H2B-mCherry. Green: α-tubulin-GFP. Cyan arrow: giant pleomorphic nucleus; green arrows: dispersed spindle assemblies; white arrow: a micronucleus. (Bottom panel) Schematic illustration of cell proliferation kinetics. The time format is hours: minutes: seconds. Bar equals 20 μm.

Here we demonstrate two subtypes of PGCCs: ones that use MEM for their nuclear growth and division (Fig. 3A) and ones that use RMEM for their nuclear growth and division (Fig. 3B).

#### MEM

As shown in Fig. 3A, panel a, two neighboring cells (marked c1 and c2) were closely attached with well-defined cytoplasmic membrane boundaries (also see Supplementary Fig. S4). Following the first mitosis of the cell c1 (Fig. 3A, panel b, white arrows), the two daughter cells (c1-a and c1-b) were internalized into their PGCC neighbor (cell c2) and formed a larger multinucleated PGCC (Fig. 3A, panel c, and Supplementary SV03-SECTION 1). Subsequently, following the nuclear envelope breakdown, all nuclei were synchronized at prometaphase (Fig. 3A, panel d). Then, a single giant chromosome wreath was assembled, suggesting that all nuclei simultaneously entered metaphase for a MEM event (Fig. 3A, panel e).

The giant nucleus further condensed itself, underwent MEM followed by karyokinesis, and split into at least six daughter nuclei (Fig. 3A, panel f). Then, the condensed chromosomes gradually relaxed and formed a multinucleated PGCC (Fig. 3A, panel g). After the first round of MEM, multiple plasma membrane pockets were observed with a fecundity cell (Fig. 3A, panel h, white arrow). Subsequently, the multinucleated PGCC underwent nuclear reorganization again and displayed a rosette and morula-like morphology (Fig. 3A, panel i). Then, over the next 120 h, the giant nucleus underwent three additional MEM events (Fig. 3A, panels j–l, second round; panels m–o, third round; panels p–r, fourth round). Notably, a few daughter nuclei became cellularized during the last endomitotic event and formed fecundity cells (Fig. 3A, panels q–r, white arrows).

The intervals between these sequential endomitotic events increased with each round of nuclear replication (~25.3 h between rounds 1 and 2; ~32.0 h between rounds 2 and 3; ~40.0 h between rounds 3 and 4; and ~66.9 h between rounds 4 and 5; Fig. 3A, bottom panel). The duration of endomitosis also increased with each endomitotic event: 2.0 h for the first one (canonical mitosis); 2.75 h for the second; 3.0 h for the third; 3.25 h for the fourth; and up to 7 h for the fifth. The giant nuclei at metaphase (endo-metaphase) were observed in phase contrast images (Supplementary Fig. S4, red arrow). In summary, MEM led to rapid nuclear augmentation by multiple rounds of synchronized nuclear replication and nuclei separation, followed by cellularization to form fecundity cells.

#### RMEM

To study how RMEM contributes to nuclear augmentation, we monitored spindle formation and nuclear dynamics in organoids labeled with H2B-mCherry and α-tubulin-GFP. The organoids were cultured on cover slides to permit the monitoring of spindles and nuclear dynamics.

A representative RMEM event is shown in Fig. 3B. Initially, a PGCC had a giant, highly pleomorphic nucleus (Fig. 3B, panel a, cyan arrow). At an early stage of RMEM, the PGCC displayed key morphologic features of prophase and prometaphase, such as a collapsed nuclear envelope with the assembly of multiple spindles (Fig. 3B, panels a–c). Multiple emerging but disorganized spindles (green arrows) were surrounded by numerous micronuclei (Fig. 3B, panel b, and Supplementary Fig. S5, white arrows). Within the next 15 min, the primary spindles further matured, as indicated by multiple hot spots showing intensive GFP fluorescence. While the giant nucleus further collapsed, the chromosomes progressively condensed. These spindles formed an irregular-shaped giant ring on top of the condensed chromosomes (Fig. 3B, panel c), indicating that the PGCC had entered metaphase. However, these poorly aligned chromosomes failed to separate (Fig. 3B, panel d) before progressing into the endo-G1 phase with relatively relaxed chromatin (Fig. 3B, panel e), suggesting a defect in anaphase. This RMEM event created a pleomorphic giant and fragmented nucleus due to disorganized spindles with poorly separated chromosomes (Fig. 3B, panel f, and Supplementary Fig. S5).

The second RMEM event occurred at ~92:00:00, where the nucleus collapsed and exhibited irregular decondensation of chromosomes with highly disorganized spindles (Fig. 3B, panels g–j). At the end of the second RMEM event, the nucleus acquired a gigantic size of 110 μm × 97 μm (long axis * short axis). The nucleus became highly pleomorphic, with numerous micronuclei scattered in the cytoplasm (Supplementary SV03-SECTION 2).

The first RMEM event lasted ~4.25 h, and the second lasted ~7.5 h (Fig. 3B, bottom panel).

### Cell cycle dynamics of MEM and RMEM

To understand the cell cycle dynamics of MEM or RMEM, we labeled the organoids with a fluorescent ubiquitination-based cell cycle indicator (FUCCI) system and subjected the cells to long-term time-lapse imaging. The FUCCI system employs a red (mKO2) and a green (mAG) fluorescent protein marker fused to two regulators of the cell cycle, -CDT1 and GEMININ. The red color indicates the G1 phase, yellow indicates the transition of G1 to S, green indicates S/G2/M, and colorless indicates later M and G0 [28, 40].

**Fig. 4:**
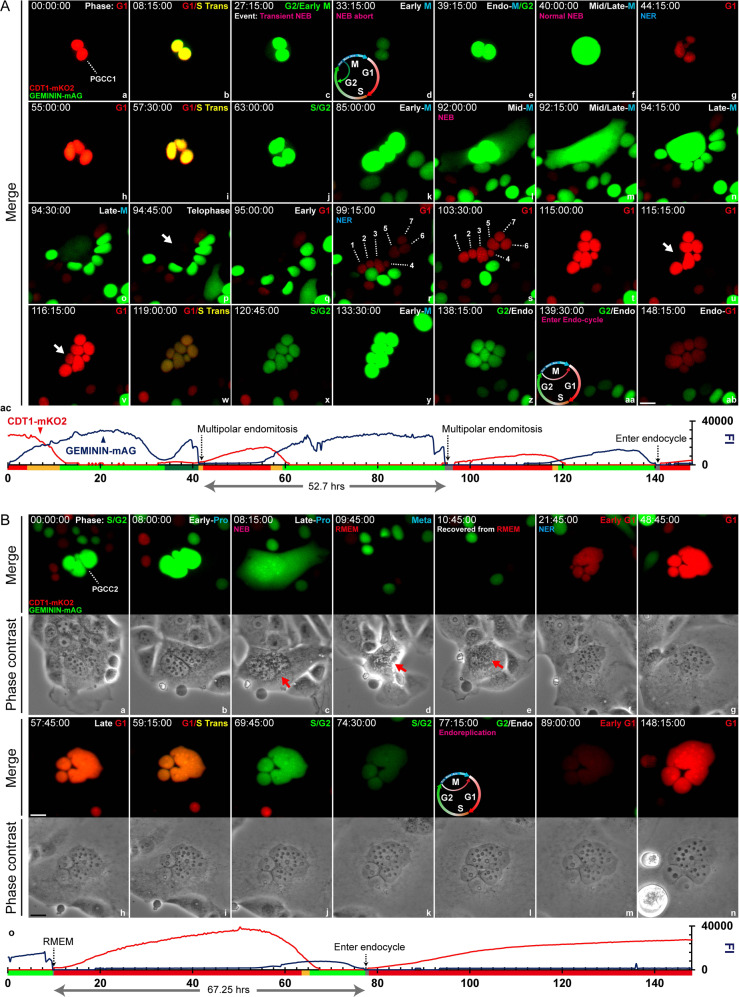
PGCC cycle dynamics of MEM and RMEM. **A** The time-series images demonstrate giant cell cycle configurations that underlie MEM. a-ab. Time-series images of FUCCI-labeled MDA-HGSC-2414 cells over approximately 148 h. G1 phase marker: CDT1-mKO2, red; S/G2/M phase marker: GEMININ-mAG, green; G1/S transition period: yellow. NEB nuclear envelope breakdown, NER nuclear envelope recovery, G1 G1 phase, G1/S Trans G1/S transition, G2 G2 phase, M metaphase. ac A line chart shows the dynamic fluctuations of the G1/G2-dependent fluorescence intensity (FI). The time format is hours: minutes: seconds. Bar equals 20 μm. **B** Time-lapse images of a PGCC underwent RMEM. a–n Time-lapse images of FUCCI-labeled MDA-HGSC-2414 cells over approximately 148 h. Phase contrast images are shown below the corresponding fluorescence images. The experimental procedures and color-rendering methods are consistent with those in panel **A**. o Same as **A**, panel ac. Bars equal 20 μm.

A representative MEM event is shown in Fig. 4A. At initial status, this PGCC (marked as PGCC1) contained two similar-sized nuclei and displayed canonical cell cycle progression from the endo-G1 phase. Then it reached the early endo-M phase, indicated by a transition of red to yellow and then green nuclei (Fig. 4A, panels a–c). Following a transitory nuclear membrane breakdown and recovery (Fig. 4A, panels d, e), the cell initiated endomitosis (Fig. 4A, panel f) and yielded three nuclei (Fig. 4A, panels g, h). Subsequently, the PGCC underwent endo-G1-S-G2-M-G1 phases and generated seven well-separated nuclei (Fig. 4A, panels i–q; individual nuclei are labeled in panels r, s).

During the progression of the endo-G1 phase, one individual nucleus momentarily lost its red fluorescence and then recovered before entering the endocycle (cell cycling between S and G phases without mitosis) with the rest of the nuclei (Fig. 4A, panels t–v, white arrows), which suggests that some nuclei can be temporally dis-synchronized during the endo-G1 phase. Then, this seven-nucleated PGCC entered an endo-S phase (Fig. 4A, panels w, x) and bypassed endomitosis (Fig. 4A, panels y, z) before starting endoreplication (Fig. 4A, panels aa-ab).

The quantitative analysis of raw images revealed multiple peaks of the GFP/RFP fluorescence fluctuations in relationship with the corresponding cell cycle events described above (Fig. 4A, panel ac). The timeline of the events is summarized in Fig. 4A, panel ac, and time-lapse video Supplementary SV04, respectively.

A representative example of RMEM is shown in Fig. 4B. At the endo-S/G2 phase, a PGCC (PGCC2) contained a cluster of irregular nuclei (Fig. 4B, panel a). Approximately 8 h later, the PGCC reached early prophase, indicated by a short expansion of nuclei with enhanced green fluorescence (Fig. 4B, panel b). However, the nuclear envelope only partially broke down with an incomplete detachment of the cell (Fig. 4B, panels c, d, red arrows). The PGCC showed completely diminished fluorescence at 09:45:00 (Fig. 4B, panel d) and then attached again (Fig. 4B, panel e, red arrow). The nuclei bypassed endo-anaphase and progressed to the next endo-G1 phase (Fig. 4B, panel f, red nuclei), endoG1/S transition (Fig. 4B, panels g–i, yellow nuclei), then entered the endo-G2/M phase (Fig. 4B, panels j, k, green nuclei), followed by complete bypass of the next endomitotic phase (Fig. 4B, panel l, colorless nuclei) before entering the next endo-G1 phase (Fig. 4B, panels m, n). The time intervals of the cell cycle dynamics from the two types of PGCCs with a diploid control are shown in Fig. 4B, panel o, Supplementary Fig. S6, and Supplementary SV04.

Our data reveal that PGCCs use highly flexible modes in cell cycle for DNA replication and nuclear augmentation after a partial or complete bypass of the mitotic phase. Depending on the specific mitotic stage that PGCCs bypass, they develop variably shaped nuclei, ranging from well-separated nuclei to highly pleomorphic giant nuclei with micronuclei-derived nuclear fragmentation.

### Decellularization, nuclear budding, and entosis

In addition, we observed three additional important modes for PGCC polyploidization and de-polyploidization in type 2 organoids: decellularization, nuclear budding, and entosis.

1. *Decellularization*. As shown in Fig. 5A, panel a, a morula-like PGCC with multiple fecundity cells (white arrows) was observed. Over time, the plasma membranes of these fecundity cells gradually dissolved (Fig. 5A, panels b–e, cyan arrows), which further fused to form a gigantic nucleus (Fig. 5A, panel f, cyan arrows, and Supplementary SV05). The corresponding phase contrast and color-coded images can be found in Supplementary Fig. S7A.

**Fig. 5:**
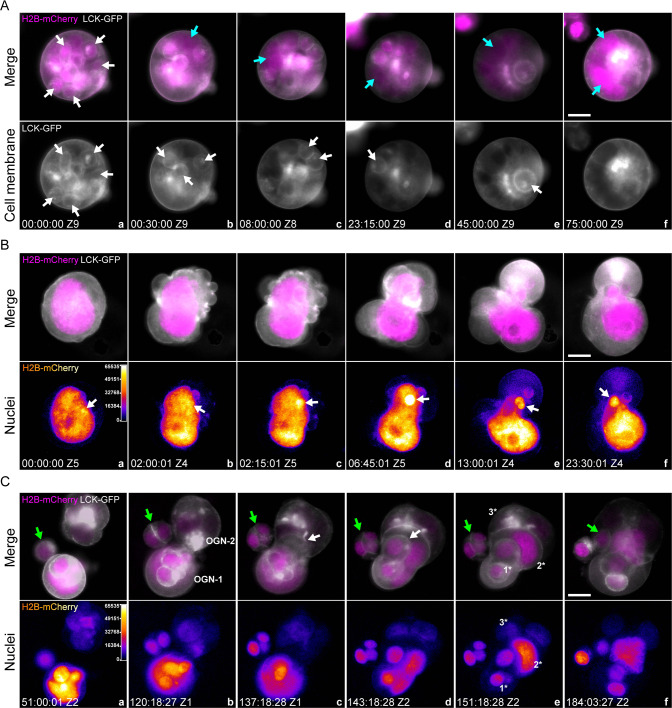
Decellularization, cellularization, and entosis of PGCCs. **A** Multiple fecundity cells in a type 2 organoid were decellularized. Gray: cell membrane, LCK-GFP. Magenta: nuclei, H2B-mCherry. Grayscale images of LCK-GFP fluorescence for visualizing the cell membrane envelopes of the host PGCC and its fecundity cells. White arrows in panel a point to a representative fecundity cell. Cyan arrows in panels b–f point to the giant nucleus generated by the released nuclei from the fecundity cells via nuclear fusion. The time format is hours: minutes: seconds. Bar equals 20 μm. **B** An amitotic mechanism (nuclear budding) generates a fecundity cell in a mononucleated PGCC. a–f Top panels, Gray: cell membrane, LCK-GFP. Magenta: nuclei, H2B-mCherry. a–f Bottom panels: Color-coded images of H2B-mCherry fluorescence. The regions of high fluorescence intensity indicate the locations of the formation of fecundity cells. White arrows: the extruding chromatin aggregate on the giant nucleus. The time format is hours: minutes: seconds. Bar equals 20 μm. **C** Time-lapse images demonstrate entosis events between two type 2 organoids. Top panels: merged fluorescence images of the cell membrane (LCK-GFP, grayscale) and nuclei (H2B-mCherry, magenta). Bottom panels: color-coded H2B-mCherry fluorescence images for monitoring nuclear status, such as condensed chromosomes. Z1 and Z2 indicate the Z-slice position of the current image. Green arrows: diploid cells gradually migrated to the organoids; white arrows: the “invasion front” of the OGN1 after infiltrating OGN2. Three prominent layers of the fecundity structure are marked by 1*, 2*, and 3*. The time format is hours: minutes: seconds. Bars equal 20 μm.
2. *Nuclear budding*. As shown in Fig. 5B, panel a, a gigantic nucleus initiated an intense reorganization within a twisted cell body. Note that a small area on the nucleus displayed higher fluorescence intensity and formed a “hot spot” (Fig. 5B, panels a, b, white arrows). The nucleus further twisted itself, extruding a small chromatin aggregate from the hot spot (Fig. 5B, panels c, d, white arrows). Multiple intracellular membrane pouches were simultaneously formed during this nuclear reorganization process (Supplementary Fig. S7B, black arrows). One of which encapsulated the extruded nucleus and formed a fecundity cell (Fig. 5B, panels e, f, white arrows; Supplementary SV06 SECTION-1). The corresponding phase contrast and color-coded images are shown in Supplementary Fig. S7B.
3. *Entosis*. Previous studies have documented that a cell can be internalized by a neighbor cell, referred to as entosis [41]. As shown in Fig. 5C, panel a, one small cell (non-PGCC, green arrow) was located close to two type 2 organoids (OGN-1 and OGN-2) made of PGCCs. At ~120:00:00, OGN-1 started migrating towards OGN-2 (Fig. 5C, panel b) and partially integrated into its framework (Fig. 5C, panel c). At 143:18:28, ~50% of OGN-1 was integrated into the OGN-2 (Fig. 5C, panels d), but the OGN-1 still showed an intact structure (white arrow, fecundity cell). By the end of this entotic event, OGN-1 and OGN-2 were reorganized to form a nested fecundity structure (Fig. 5C, panels d, e, and Supplementary SV06 SECTION-2). Of note, a progeny of the small cell was also internalized by OGN-2 and incorporated into this Russell doll-like multi-layer fecundity structure (Fig. 5C, panels a–f, green arrows).

### Distinct proliferation kinetics in type 1 and type 2 organoids

**Fig. 6:**
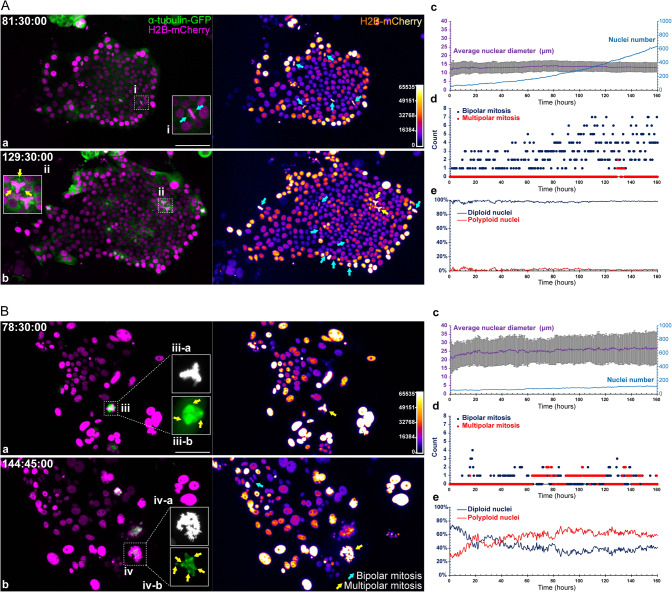
Cell proliferation kinetics sustaining the growth of type 1 and type 2 organoids. **A** Cell proliferation kinetics of a type 1 organoid with minimal PGCCs. a, b (left panels) spindles (α-tubulin-GFP, green) and nuclei (H2B-mCherry, magenta). a, b (right panels) Corresponding color-coded images for H2B-mCherry fluorescence, indicating relative DNA contents. Insets i show typical mitotic figures of canonical mitosis. Inset ii shows tripolar open mitosis (cyan arrows). The time format is hours: minutes: seconds. Bar equals 100 μm. c–e Quantitative analyses from the time-lapse images. c Dynamic change in average nuclear diameter (left y-axis) and absolute nuclear number (right y-axis). The data represent the mean ± S.D. for the average nuclear diameter values. d Distribution of the mitotic event incidence (the numbers of all observed mitotic events, including bipolar and multipolar types, on the analyzed image) during the expansion of the cell colony. e Percentages of diploid and polyploid nuclei during organoid growth. **B** Cell proliferation kinetics of a type 2 organoid with abundant PGCCs. The composition, layout, and measurements are consistent with **A**. Inset iii series represents a tripolar mitotic event; inset iv series represent a multipolar mitotic event. The suffix **-**a indicates the H2B-mCherry fluorescence (grayscale), and the suffix -b indicates the α-tubulin-GFP fluorescence (green).

To understand the difference in growth kinetics between type 1 and type 2 organoids, we cultured the organoids on slides to allow identification and counting of the mitotic figures. As shown in Fig. 6A, a typical type 1 organoid showed uniform nuclei after attachment. With the expansion of the cell colony, most cells predominantly relied on traditional mitosis for growth, as indicated by the high frequency of bipolar (Fig. 6A, pane a, inset i, cyan arrows) or occasional tripolar open mitotic figures (Fig. 6A, pane b, inset ii, yellow arrows).

In contrast, a typical type 2 organoid displayed highly pleomorphic nuclear morphology with an increased frequency of multipolar endomitosis (Fig. 6B, pane a, insets iii-a and iii-b, yellow arrows). More atypical mitotic events were observed along with the expansion of colony size, including highly disorganized MEM at 144:45:00 (Fig. 6B, insets iv-a and iv-b). Color-coded images of histone H2B-mCherry fluorescence suggested the gradually increased number of polyploid nuclei and DNA contents.

The corresponding quantitative data of the above organoids are summarized in Fig. 6A, panels c–e, and Fig. 6B, panels c–e. Three distinctive features were observed between the two types of organoids.

First, the type 1 organoid displayed relatively small but more uniform nuclei within a range of 10–15 μm in diameter (on 2D condition) (Fig. 6A, panel c), while the type 2 organoid displayed notably increased nuclear diameter (15–35 μm) (Fig. 6B, panel c).

Second, the type 1 organoid employed canonical mitosis for its growth and division, resulting in a logarithmic increment of cell numbers (Fig. 6A, panels c, d). In contrast, the type 2 organoid displayed a slow increment of cell number with a higher incidence of multipolar endomitosis (Fig. 6B, panels c, d).

Third, the type 1 organoid contained minimal polyploid nuclei (less than 5% on average), while the type 2 organoid displayed 20–60% polyploid nuclei, increasing with time (Fig. 6B, panel e, vs. Fig. 6A, panel e). The progressive increment of polyploid nuclei can be observed in the time-lapse video Supplementary SV07.

### PGCCs and fecundity cells in HGSCs

**Fig. 7:**
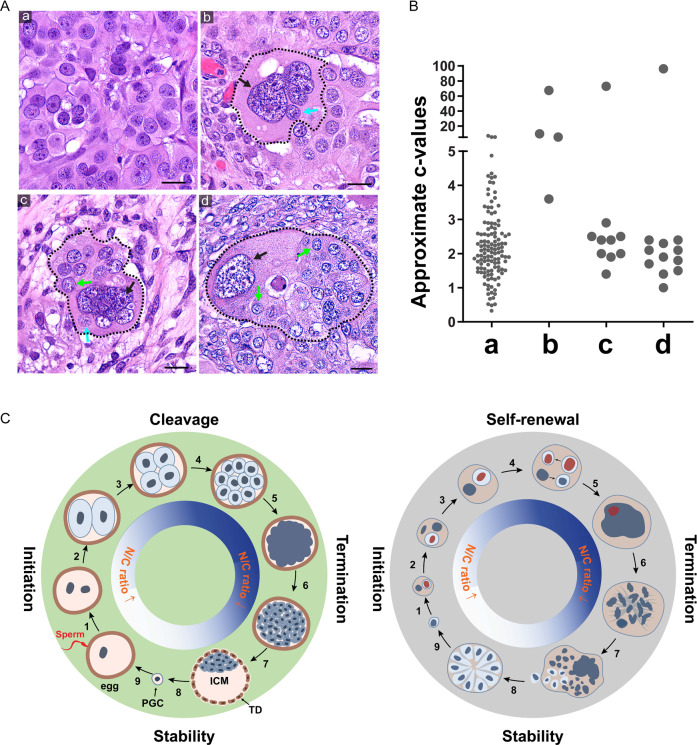
The fecundity structures in patient HGSC tumors. **A** Patient tumor tissues contain histologic structures similar to the type 1 and type 2 organoids. a type 1 organoid-like tissue structure. b–d Type 2 organoid-like tissue structure at different stages of the giant nucleus with partially or entirely cellularized small nucleus. Black arrows: giant nuclei of the host cells. Cyan arrows: fecundity cells. Green arrows: cell colonies formed by the released fecundity cells. Bars equal 20 μm. **B** The estimated c-value (DNA content) of the nuclei showing in **A**. The measurements of the group a were performed with an uncropped image. The measurements for groups b–d include the nuclei within the areas confined by the dotted lines. The ploidy level was calculated based on the average nuclear sizes following the formula for the volume of an ellipsoid: (Vn = (4/3)*pi*X*Y*Z), where X is the long axis of the nucleus. Ploidy level was calculated following the formula c = 2*(Vx/Vd), where Vx denotes the volume of the nucleus to be measured, and Vd denotes the average nuclear volume of the reference diploid cells. **C** Schematic of pre-implantation pre-embryogenesis and the life cycle of PGCC. Left panel Illustration of pre-embryogenesis in the pre-implantation embryo. Fertilization initiates nuclear cleavage (1) to generate smaller-sized blastomeres with decreased cell size and increased nuclear-to-cytoplasmic ratio (2–4). The blastomeres lose cell boundary and compact (5), become re-cellularized and form a morula (6) and further develop into a blastocyst (7). A subset of the embryoblast is set aside to develop into primordial germ cells (8) to develop into the giant egg and tiny sperm (9). The fusion of sperm with egg initiates a new round life cycle. Right Panel. The life cycle of PGCCs and cancer macroevolution. Diploid/aneuploid cells undergo asymmetric mitosis (1) and form a fecundity cell (2). The nucleus continues intracellular nuclear and cell division and nuclear fusion (3-4) and forms a giant nucleus (5), which undergoes MEM or RMEM (6,7); a subset of fecundity cells bud out, assume mitotic division, and form blastula-like organoids for microevolution (8) or resume the new life cycle of PGCCs to facilitate cancer macroevolution (9). ICM inner cell mass, N/C ratio nuclear-to-cytoplasmic ratio, PGC primordial germ cell, TP trophectoderm. The red nuclei indicate asymmetric mitosis and fecundity cell.

To further validate the above-described organoid developmental properties, we studied H&E-stained slides from HGSCs. Fig. 7A, panel a, displays a type 1-like tissue structure with well-cellularized nuclei. Fig. 7A, panels b–d, show typical type 2-like tissue structures. The black dashed lines outline the PGCCs with their fecundity cells, and the black arrows point to the giant nuclei of the host PGCCs. Panel b shows a PGCC with a giant nucleus (black arrow) with amitotic budding (cyan arrow). Panel c shows a primitive fecundity cell-based tissue structure with multiple fecundity cell nuclei (green arrow). Panel d shows a giant nucleus adjacent to multiple partially or completely cellularized fecundity cells to start to form type 1-like tissue (green arrows).

The estimated ploidy levels of the cells shown in Fig. 7A are summarized in Fig. 7B. Other typical examples of the Russian doll-like fecundity structures observed in patient tumors are presented in Supplementary Fig. S8.

## Discussion

This paper provides the first detailed spatiotemporal analysis of how a single cell generates HGSC organoids. Our study revealed a variety of novel modes of nuclear growth and division to achieve an increased N/C ratio without cell division similar to that in pre-embryogenesis, which has several implications for our understanding of malignant tumor development and cancer macroevolution.

### Resolution of a long-standing paradox

Pathologists such as Muller, Virchow, and von Hansemann, as well as the embryologist Boveri, recognized tumor morphological heterogeneity and nuclear atypia in their pioneering studies in the 19th century [1, 2, 42, 43]. However, the conventional belief in cell biology was that PGCCs were senescent cells prone to die and thus not essential. The discordance between pathologic observations of PGCCs and conventional beliefs leads to a big paradox in cancer biology: If PGCCs are nonviable, how can they contribute to the nuclear features that pathologists use to define a tumor as malignant?

Here we provide live-cell imaging evidence of the spatiotemporal nuclear dynamics of PGCCs in malignant histogenesis. The nuclei in PGCCs can replicate via various modes of nuclear replication, through endo-cycling, REM, RMEM, nuclear fragmentation, micronuclei or nuclear fusion, and divide via budding without cytokinesis or fecundity cell formation, thus providing the nuclear and cellular basis for tissue heterogeneity. By defining these novel modes of cell division, our work has thus resolved this more than century-old paradox in diagnostic pathology and cancer biology.

### The cellular and nuclear basis for cancer evolution

Traditionally, cancer has been considered a disease of uncontrolled proliferation via activation of oncogenes or loss of tumor suppressor genes that leads to dysregulated mitotic growth [44]. Gene mutations or translocations, commonly referred to as Darwinian microevolution, lead to gradual change over time to generate intratumoral heterogeneity [45–47]. The growth pattern in type 1 organoids, which leads to a relatively well-cellularized homogeneous tissue structure, is likely to be associated with microevolution. On the other hand, the formation of PGCCs that led to the formation of type 2 organoids and poorly cellularized/differentiated cancer tissue represents a stress-induced mechanism that leads to tolerance of massive genomic errors and aneuploids and facilitates the creation of a new biological system [44, 45]. The growth of type 2 organoids provides a cellular and nuclear basis for cancer macroevolution via a punctuated massive genomic structural change through the formation of a polyploid genome [44, 45, 47–50]. Thus, all these seemingly “strange” ways of nuclear replication, fusion, fragmentation, micronuclei, or entosis in PGCCs have unified genomic consequences: creating altered genome systems ready for cancer macroevolutionary selection followed by activation of molecular pathways to facilitate subclonal expansion for microevolution. These two developmental pathways works hand-in-hand for cancer genome evolution during initiation, treatment resistance, and metastasis [44, 51, 52].

### Fecundity cells and fecundity structures: a non-genetic mechanism immune escape?

Despite exciting but limited success in the subset of human cancers, it becomes clear that most solid tumors are not sensitive to commonly used immune therapy. One of the significant unsolved puzzles is how these tumors escape from immune surveillance and confer resistance to common immune checkpoint inhibitors. Transient cell-in-cell formation has been recently reported to confer resistance to immune therapy [53]. The presence of fecundity cells and Russian doll-like fecundity tissue structures may provide a novel mechanism for the observed resistance. Other groups have also observed cell-in-cell formation [41, 54] and chemotherapy-induced senescent cancer cells engulfing other cells [55]. Rather than separating into individual cells via energy-consuming canonical mitosis and cytokinesis, such rapid cellularization inside a PGCC by creating fecundity cells or fecundity structures provides a non-genetic physical mechanism to prevent immune cell infiltration. The rapid “eating” of one PGCC by another PGCC carrying a fecundity structure may confer additional physical barriers resulting in resistance to immune therapy.

### Rapid nuclear augmentation to increase N/C ratio: an evolutionarily conserved mechanism mimicking the development of pre-embryogenesis?

Previously, we showed that PGCCs recapitulated a senescence phenotype and blastomere-like pre-embryonic program, which led to massive karyotypes in PGCC-derived daughter cells for chemotherapy resistance [27, 28]. The different nuclear augmentation mechanisms that lead to an increase in the N/C ratio described in this study support our early hypothesis that the life cycle of PGCC mimics that of human pre-embryogenesis [27, 28, 56–58]. The rapid nuclear augmentation followed by cellularization in the blastula stage is observed in humans, *Drosophila*, and other species; successful nuclear division without accompanying cytokinesis is followed by cellularization at the blastula-formation stage referred to as the coenocyte [59]. Thus, these data suggest malignant histogenesis may have recapitulated an evolutionarily conserved archaic program for pre-embryonic development [57–60].

A schematic diagram of pre-implantation embryogenesis and malignant tumorigenesis is shown in Fig. 7C, left panel. Following fertilization, the zygote undergoes rapid nuclear division to increase the N/C ratio, which leads to the transformation of the zygotic genome into an early embryonic genome [60]. Starting with 1-cell embryo, there is massive incomplete replication fork stalling during cleavage division that leads to chromosomal breaks and aneuploidy [61]. The cleaved blastomeres are genomically unstable and often associated with polyploidy, aneuploidy, endoreplication, nuclear fragmentation, multinucleation, and chromothripsis [62–64]. The blastomere forms a mononucleated cell-like giant cell (compaction) and then differentiates into a multinucleated cell-like morula (termination) and subsequently develops into a blastocyst composed of trophectoderm and inner cell mass. Trophectoderm develops a genomically unstable placenta, while the inner cell mass develops into inner cell mass for embryogenesis (stability). A subset of epiblasts was set aside to develop into egg or sperm cells at puberty. If fertilization occurs, a new life cycle starts.

The life cycle of PGCC responsible for the malignant histogenesis of HGSC is shown in Fig. 7C, right panel. During initiation, following canonical mitotic division, one nucleus continues nuclear augmentation to increase the N/C ratio. During the self-renewal phase, a giant nucleus can undergo multiple rounds of the endomitotic cycle to increase the nuclear contents to facilitate macroevolution via an increased N/C ratio. During termination, the endomitotic nuclei undergo partial or complete karyokinesis and cellularization to form fecundity cells. The fecundity cells can decellularize themselves to increase the nuclear contents further. During the stability phase, the newly formed fecundity cells bud out and resume mitosis to facilitate microevolution and stable tumor growth. A subset of cancer cells can reenter the giant cell life cycle and initiate a new round of nuclear augmentation for macroevolution.

The lack of trophectoderm differentiation may be one of the key mechanisms of how malignant tumorigenesis differs from embryogenesis: in normal development, during the blastocyst formation, the unstable genomes generated during cleavage segregate into trophectoderm for the development of the placenta [57, 58]. While in the giant cell life cycle, PGCCs continuously generate and tolerate chaotic genomes for macroevolution and development in the absence of an activated differentiation program.

### Summary

Our data demonstrated that high-grade tumor development is remarkably similar to pre-embryogenic development. Continuous cycling between macroevolution and microevolution via the polyploid and diploid genomes creates a highly dynamic evolving system for malignant tumor growth in response to intrinsic and environmental stresses.

## Materials and methods

### 3D culture of organoids

HGSC organoids were cultured in 30% (v/v) Matrigel matrix (354230, Corning) mixed with serum-free culture medium prepared in our laboratory. A modified medium formulated using FluoroBrite DMEM was applied for long-term imaging. The organoids were maintained at 37 °C in a 5% CO_2_ humidified incubator/imaging chamber.

### Lentivirus packaging

The following lentiviral plasmids were purchased from Addgene: pBOB-LCK-GFP (RRID: Addgene_118738); pLenti6-H2B-mCherry (RRID: Addgene_89766); L304-EGFP-Tubulin-WT (RRID: Addgene_64060); pBOB-EF1-FastFUCCI-Puro (RRID: Addgene_86849). The second-generation packing system, including psPAX2 (RRID: Addgene_12260) and pMD2.G (RRID: Addgene_12259), was applied to produce pseudoviral particles.

### Fluorescence time-lapse imaging

For 3D imaging, the HGSC organoids were embedded in 45% phenol red–free Matrigel matrix (356231, Corning) mixed with 55% advanced imaging medium on a glass-bottom culture plate (0.16–0.19 mm thick) (801004, NEST). For a six-well plate, 4 ml of advanced imaging medium was added to each well to support organoid growth during imaging.

A Lionheart (Biotek) multifunction imaging instrument was used to perform long-term time-lapse live-cell imaging. The key optical components include DAPI cube assembly (LED: 1225007/Filter Cube: 1225100); GFP cube assembly (LED: 1225001/Filter Cube: 1225101); GFP cube assembly (LED: /Filter Cube:); Texas Red cube assembly (LED: 1225002/Filter Cube: 1225102).

Gen5+ software was applied for image acquisition and instrument control. The raw images were further processed in Fiji software (version 1.52p) (RRID: SCR_002285), Adobe Photoshop (RRID: SCR_014199), Adobe Illustrator (RRID: SCR_010279), and Adobe Premiere Pro (RRID: SCR_021315), for generating time-lapse videos and figures.

## Supplementary information

Supplemental Material

SV01

SV02

SV03

SV04

SV05

SV06

SV07

## Data Availability

This study contains no publicly available datasets or computing codes. The uncompressed video files are available upon request.

## Material availability

The human HGSC organoids included in this study are established following the ethical policies and protocols approved by The University of Texas, MD Anderson Cancer Center. All patient-derived biological materials in this study are considered unique materials which are available for non-profit studies upon request.
