# Supplementary material for: Spatiotemporal view of malignant histogenesis and macroevolution via formation of polyploid giant cancer cells
Source: Oncogene. 2023 Jan 3;42(9):665–78. doi: 10.1038/s41388-022-02588-0 (PMC9957731; doi:10.1038/s41388-022-02588-0)
Supplement: Supplementary file 1 — Supplemental Material [file 41388_2022_2588_MOESM1_ESM.docx]

**Supplementary Figures and Figure Legends**

**
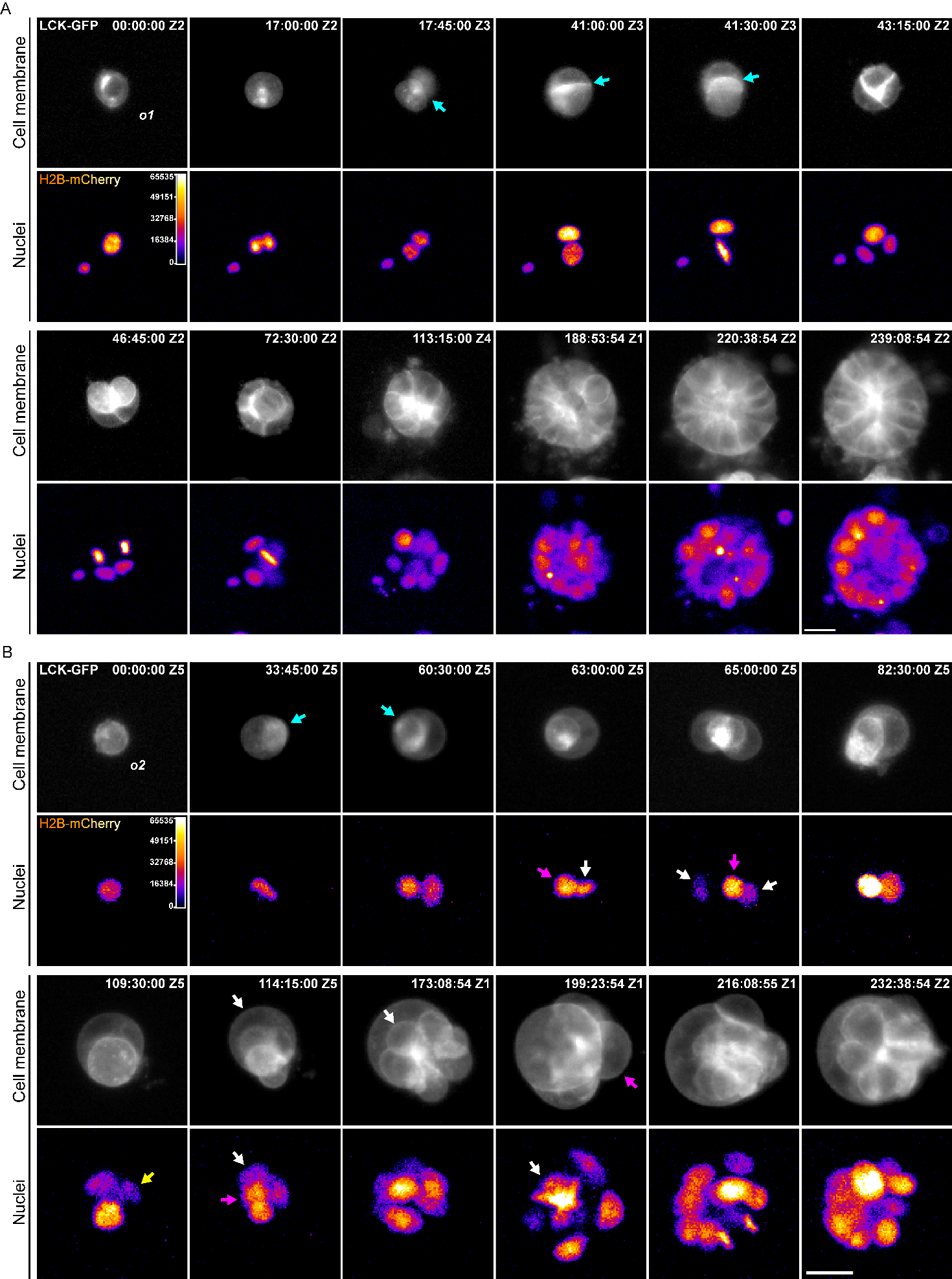
Figure S1**

Spatiotemporal view of development from a single cell to a multicellular organoid. Grey: cell membrane, LCK-GFP. Fire (color-coded): nuclei, H2B-mCherry. Images in panels A and B correspond to Figures 2A and 2B, respectively.

A. Development of a type 1 organoid. Cyan arrows: cell membrane boundary between the two daughter cells after the first round of mitosis.

B. Development of a type 2 organoid. (33:45:00, 60:30:00): cyan arrows, a polarized region on the cell membrane. (63:00:00, 65:00:00): white arrows, host PGCC; magenta arrows, fecundity cell. (109:30:00): yellow arrow, second fecundity cell. (114:15:00): white arrows, host PGCC; magenta arrow, fecundity cell. (173:08:54): white arrow, fecundity cell cluster. (199:23:54): magenta arrow, fecundity cell released at metaphase and its daughter cells; white arrow, giant chromosome assembly at metaphase in the host PGCC.

The time format is hours: minutes: seconds. Bars equal 20 μm.

**Figure S2**

**
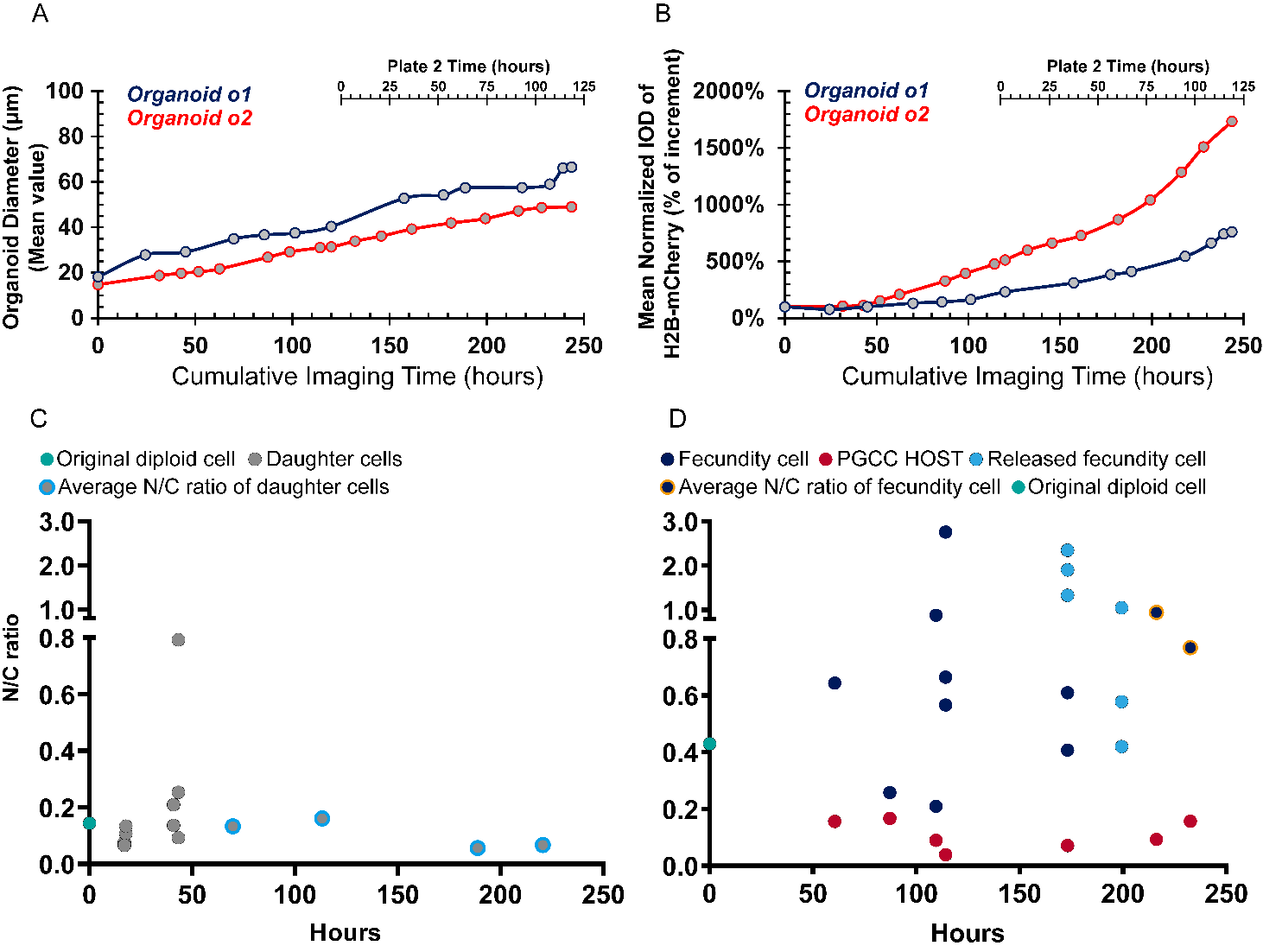
**

Change in size and genomic contents of organoids over time. A. Distinct growth patterns of type 1 (o1) and type 2 (o2) organoids based on the measurements of organoid diameters (based on LCK-GFP fluorescence, unit: μm) and B. estimates of genomic contents (based on the Integrated Optical Density [IOD] of H2B-mCherry fluorescence). C-D. The estimated N/C ratio values. For cells containing no fecundity cells, the N/C ratio = V_n_/(V_c_-V_n_), where V_n_ denotes the nuclear volume and V_c_ denotes the cytoplasmic volume. V_n_ and V_c_ are calculated using the ellipsoid formula: V=(4/3)*π*(a*b*c), where a, b, and c are radii in the x, y, and z axes. For cells containing fecundity cells, the N/C ratio = V_nPGCC_/(V_cPGCC_-V_fcs_-V_nPGCC_), where V_nPGCC_ denotes the cytoplasmic volume of the PGCC; V_fcs_ denotes the total volume of all fecundity cells; and V_nPGCC_ denotes the volume of the nuclei of the giant host PGCC.

**Figure S3**


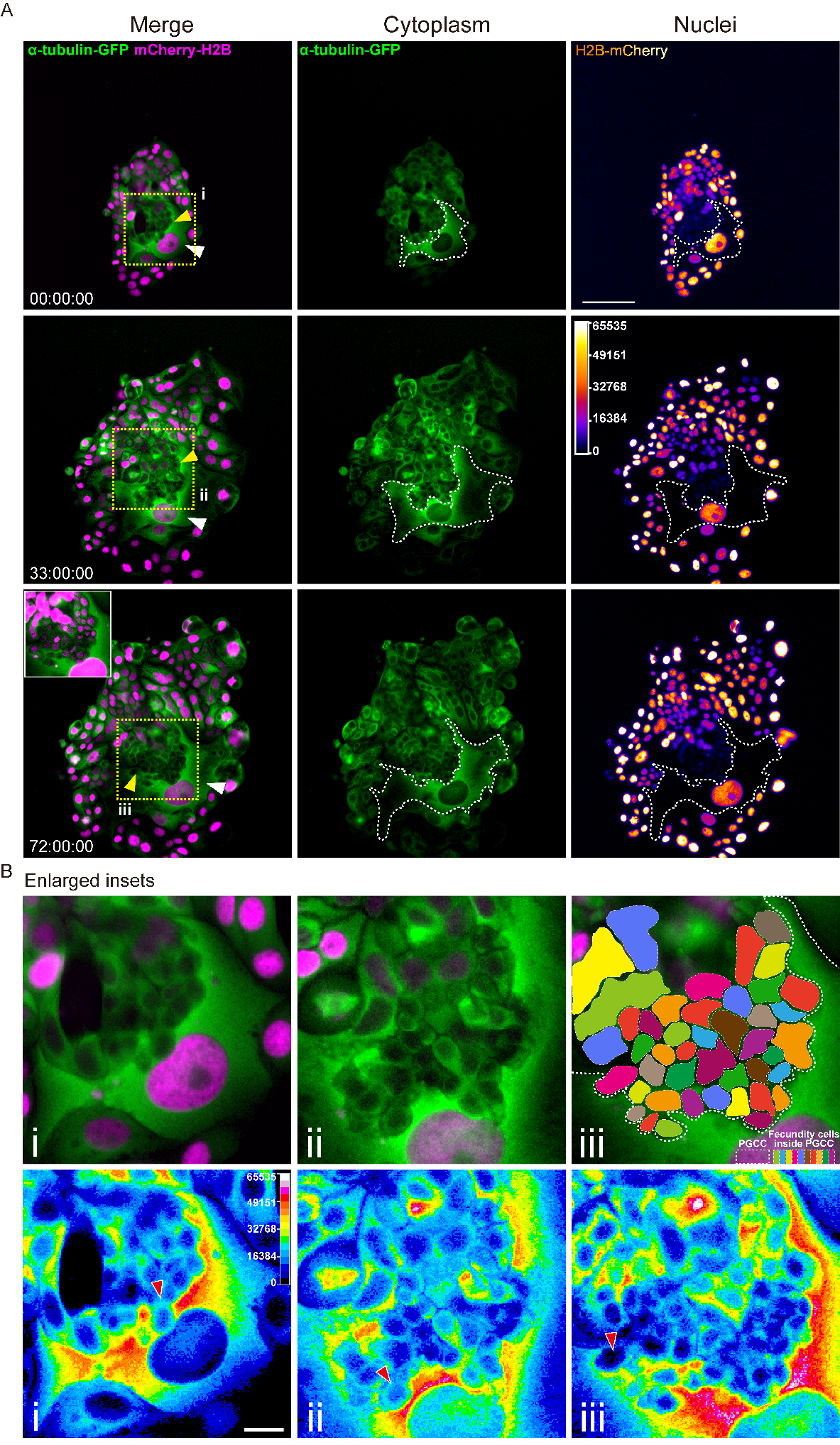


High proliferation activity of the fecundity cells released from an MDA-HGSC-2414 type 2 organoid. The organoid was selected under a fluorescence microscope and transferred to cover glasses for long-term culture. Once the organoids were attached to the glass, time-lapse fluorescence imaging was initiated. Green, α-tubulin-GFP; Magenta, H2B-mCherry

A: Fecundity cell growth within mother PGCCs at 0 hours, 33 hours, and 72 hours. Left panels: Merged time-lapse images of α-tubulin-GFP (green) and H2B-mCherry (magenta) fluorescence. Yellow arrowheads: fecundity cells; white arrowheads: cell body of the host PGCC (indicated by enhanced α-tubulin-GFP fluorescence). Dotted lines outline the borders of the host PGCC. In the lower-left panel, the inset shows an enhanced image of the area confined by the dotted lines to observe the clustered fecundity cells; Middle panels: fluorescence images of α-tubulin-GFP (green); Right panels: color-coded images of H2B-mCherry fluorescence demonstrate the nuclear number, distribution, and fluorescence intensities.

B. Upper panels: enlarged areas corresponded to insets i, ii, and iii in the left panel above. In the upper-right panel, the fecundity cells were highlighted by colored masks. Lower panels: color-coded images of the fecundity cells based on the α-tubulin-GFP fluorescence from insets i, ii, and iii. Note the increased numbers of ovals contrasted by the GFP fluorescence, indicating proliferating fecundity cells. The red arrowheads point to three representative fecundity cells. The time format is hours: minutes: seconds. Bar equals 100 μm.

**
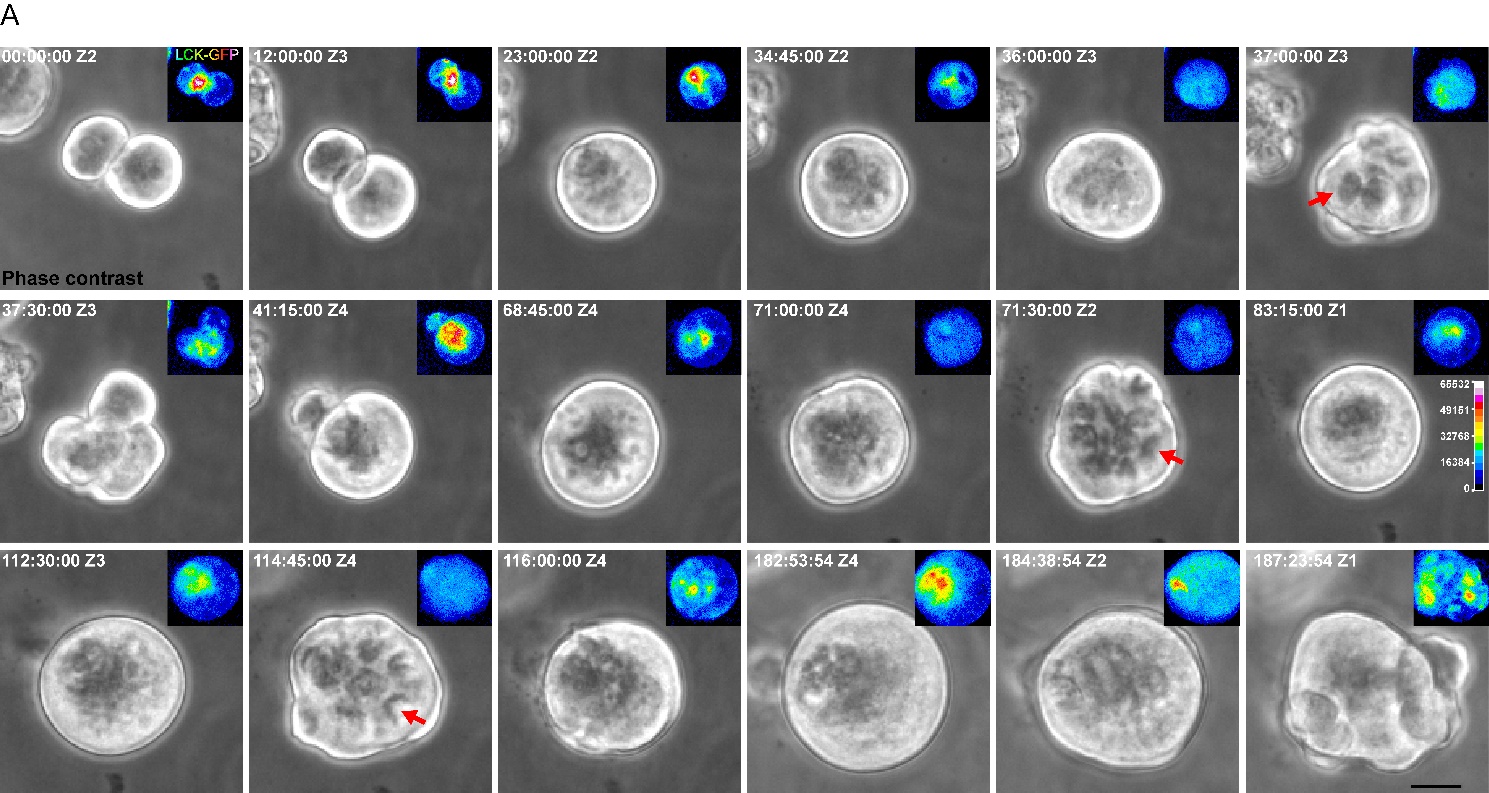
Figure S4**

Time-lapse images, including phase contrast and color-coded LCK-GFP (cell membrane) fluorescence images, correspond to Figure 3A. Note that the giant chromosome assemblies are visible without any fluorescence labeling at 37:00:00, 73:30:00, and 114:45:00, marked by red arrows. The time format is hours: minutes: seconds. Bar equals 20 μm.


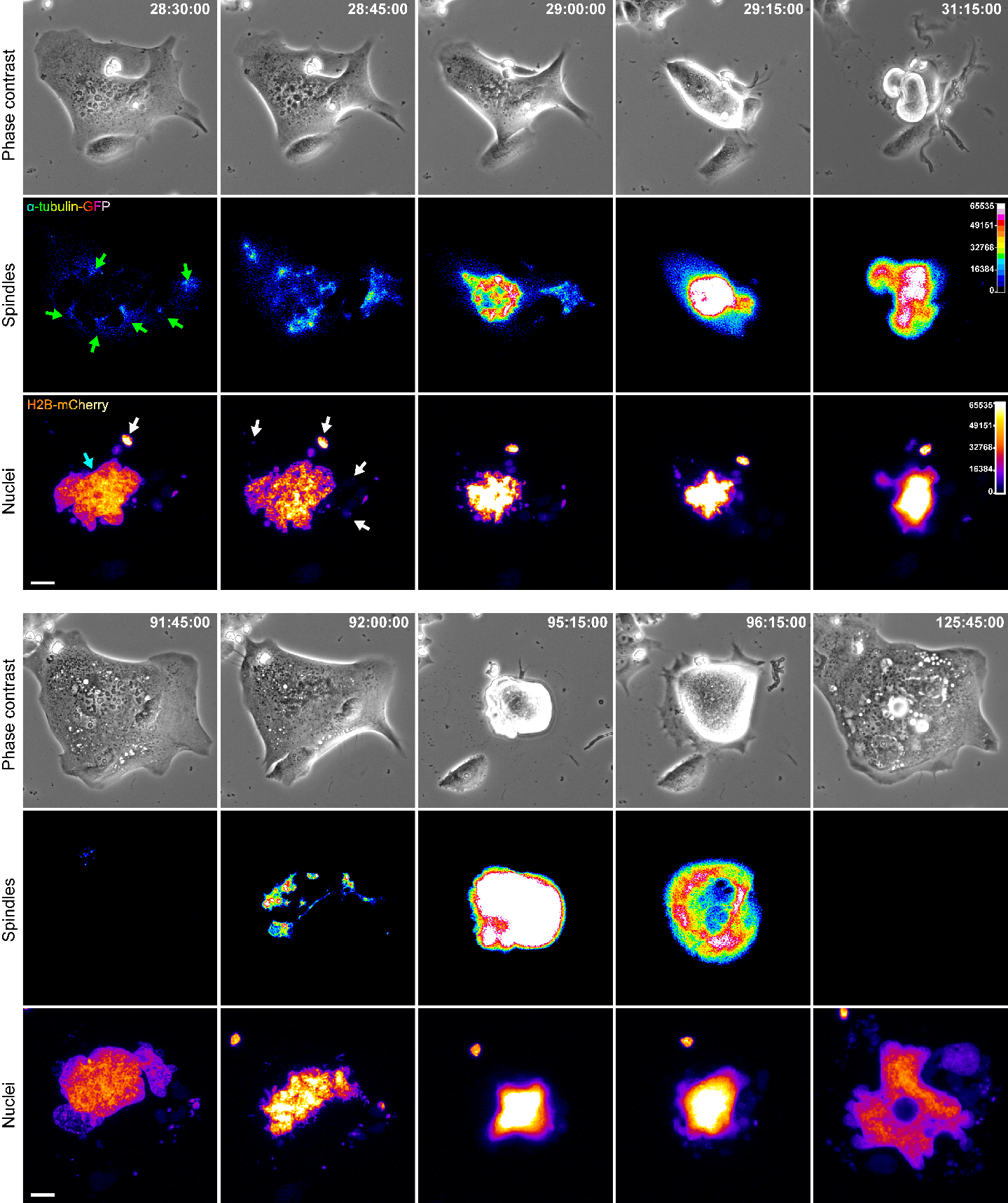
**Figure S5**

Time-series images, including additional phase contrast and color-coded LCK-GFP (cell membrane) and H2B-mCherry (nuclei) fluorescence images corresponding to Figure 3B. Green arrows: scattered spindle assembly; cyan arrow: polyploid giant nucleus; white arrows: micronuclei. The time format is hours: minutes: seconds. Bars equal 20 μm.

**Figure S6**


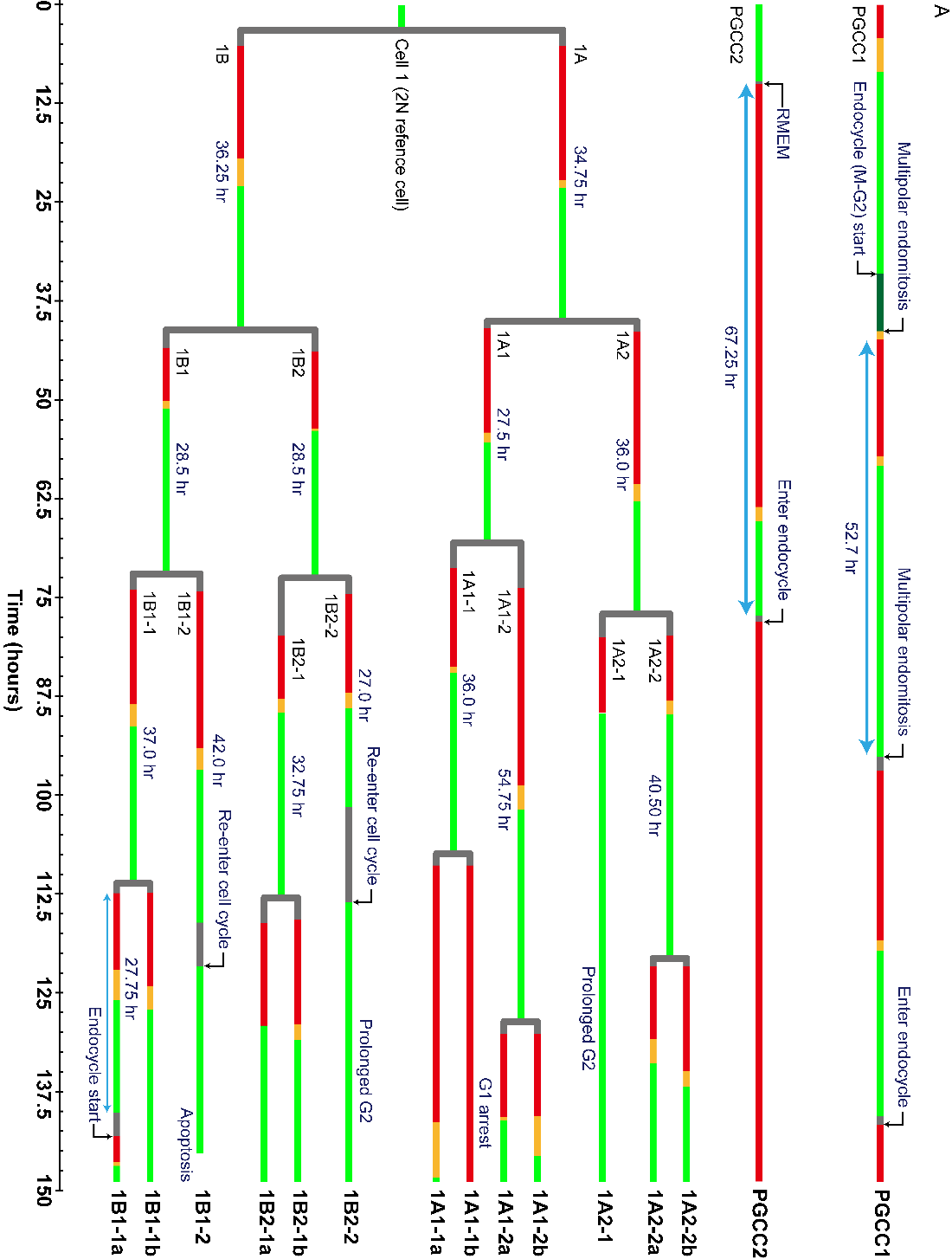


Schematic of the cell cycle configurations of the two PGCCs described in Figures 4A and B, with a continuously dividing diploid reference cell, including its progeny cells. This diploid reference cell is labeled cell 1, and the progeny cells are labeled with suffixes to indicate their generation. Red: G1 phase; yellow: G1/S transition; green: late S/G2/early M; gray: colorless (post-M or G0).

**Figure S7**

**
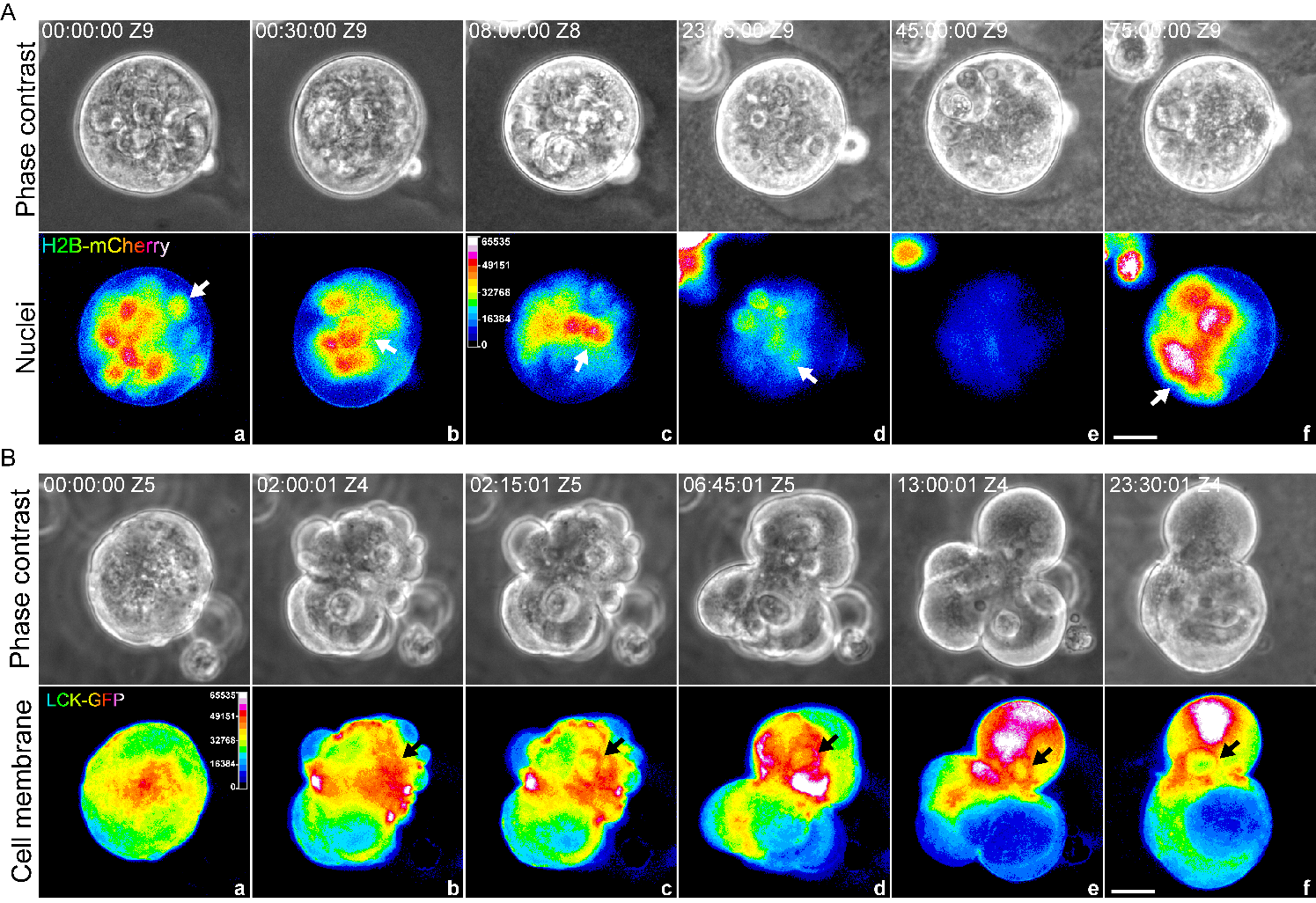
**

A. (a-f) Phase contrast (top) and color-coded H2B-mCherry fluorescence (bottom) images correspond to Figure 5A. The fecundity cells can be seen as complex intracellular structures at earlier stages but were gradually dissolved with the re-formation of the PGCC. White arrows: Individual nuclei progressively fused into a giant nucleus.

B. (a-f) Phase contrast (top) and color-coded LCK-GFP fluorescence (bottom) images correspond to Figure 5B. Black arrows: Plasma membrane envelope that wrapped the extruded chromatin aggregate (budding nucleus). The images demonstrate an active restructuring process during fecundity cell formation via nuclear budding (amitotic pathway).

**Figure S8**


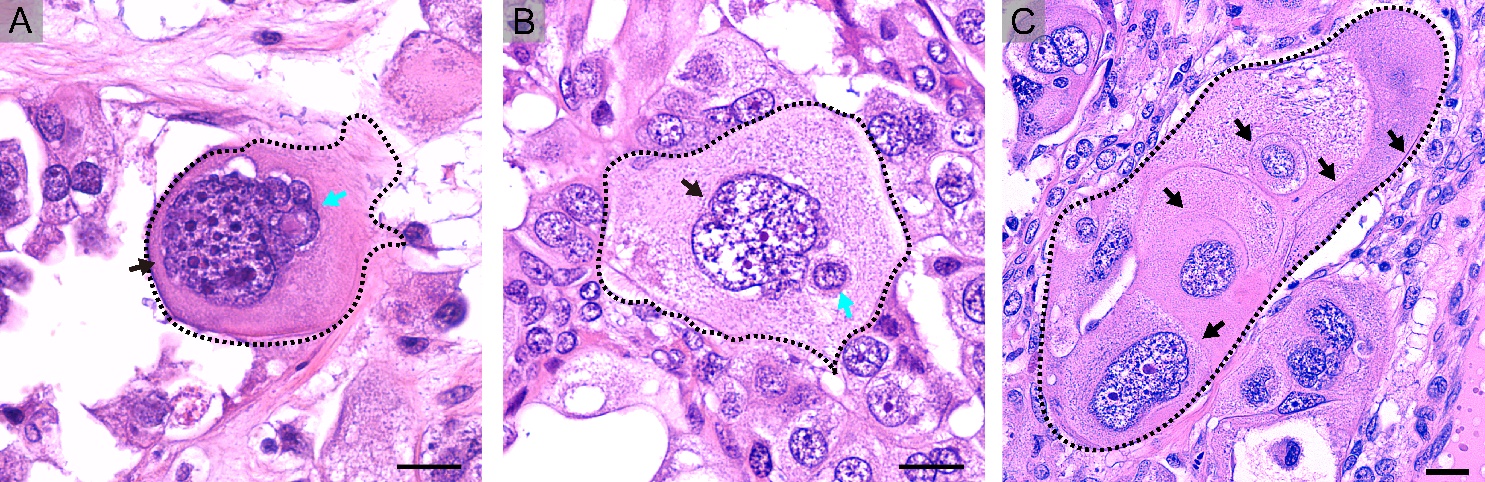


Patient tumors contain fecundity structures similar to those observed in organoid studies. A. Early nuclear budding. Black arrow: giant nucleus of the PGCC; cyan arrow: early budding daughter nuclei. B. A primitive form of fecundity structure. Black arrow: giant nucleus of the PGCC; cyan arrow: a fecundity cell. C. An example of a Russian doll-like fecundity structure that contains at least five layers of fecundity cells. Black arrows: The cell boundary of the five fecundity structures.

**Video Legends**

For the videos attached to this paper, the following general rules apply unless indicated otherwise:

1. LCK-GFP fluorescence indicates the position of the cell membrane.
2. H2B-mCherry fluorescence indicates the position of the cell nucleus/nuclei.
3. α-tubulin-GFP fluorescence indicates the position of the spindle and indicates mitotic events.
4. Labels consisting of "P" followed by a number denote plate numbers, which indicate the sequential image sets generated by a program that allows the user to pause the imaging program for medium change. When the imaging program is set back to live, the captured images are stored in a new dataset. The first image dataset is labeled P1; the second is labeled P2.
5. Each channel's relative fluorescence intensity (16-bit depth) is indicated by the scale meter shown in the corresponding panel.
6. The Z numbers indicate the Z-axis position of the current slice in the stack, e.g., Z2 means the second image in the stack.
7. FPS is the abbreviation of frames per second.

***SV-01***

**SV01-SECTION 1**

Representative type 2 MDA-HGSC-2414 HGSC organoid with fecundity cells captured by laser confocal microscopy with Z-axis scanning. The animation was generated from 3D reconstructed images rendered with multiple color-coding methods. Top left: LCK-GFP, green, 70% transparency; H2B-mCherry, magenta. Top right: LCK-GFP, green, 50% transparency; H2B-mCherry, magenta. Lower left: LCK-GFP, color-coded. Lower right: H2B-mCherry, color-coded.

**SV01-SECTION 2**

Representative type 2 MDA-HGSC-2414 HGSC organoid with fecundity cells captured by laser confocal microscopy with Z-axis scanning. This video shows a simultaneous playback of the 3D reconstructed images of the cell membrane structure (LCK-GFP, color-coded) and nuclei (H2B-mCherry, color-coded) with Z-axis scanning animation. Bar equals 20 μm.

***SV-02***

**SV02-SECTION 1**

MDA-HGSC-2414 organoids were disassociated into single cells and then embedded in a phenol red-free Matrigel matrix. This video demonstrates a typical process of development of a well-cellularized HGSC organoid (type 1) from a single cell. The frame rate for coding the uncompressed video file was 10 FPS. The time interval between each frame is 15 minutes. Top left: merged images of LCK-GFP, gray; H2B-mCherry, magenta. Top right: phase-contrast, gray; Lower left: H2B-mCherry, color-coded. Lower right: LCK-GFP, color-coded. Bar equals 50 μm.

**SV02-SECTION 2:**

This video demonstrates a typical development process of a poorly cellularized HGSC organoid (type 2) from a single cell. The composition and rendering methods are consistent with those for SV02-SECTION 1.

***SV-03***

**SV03-SECTION 1**

This video demonstrates a PGCC showing multiple multipolar endomitosis (MEM) events during its proliferation under a 3D culture condition. Top left: merged images of phase-contrast, gray; H2B-mCherry, magenta. Top right: merged images of LCK-GFP, gray; H2B-mCherry, magenta. Lower left: LCK-GFP, color-coded. Lower right: H2B-mCherry, color-coded. The time interval between each frame is 15 minutes. The frame rate for coding the uncompressed video file is 10 FPS. Bar equals 50 μm.

**SV03-SECTION 2**

This video demonstrates a PGCC showing restitution multipolar endomitosis (RMEM) under a 2D culture condition. Top left: merged images of phase-contrast, gray; H2B-mCherry, red. Top right: merged images of α-tubulin-GFP, green; H2B-mCherry, magenta. Lower left: α-tubulin-GFP, color-coded. Lower right: H2B-mCherry, color-coded. Bar equals 50 μm. The time interval between each frame is 15 minutes. The frame rate for coding the uncompressed video file was 5 FPS.

***SV-04***

This video demonstrates the differences in cell cycle kinetics between the canonical mitotic cycle, MEM, and RMEM. The red of CDT1-mKO2 indicates the G1 phase; the green of GEMININ-mAG indicates the S/G2/M phases, and the yellow is generated by mixing red and green and indicates the G1/S transition. Top left: Merged fluorescence video of diploid cells for demonstrating canonical mitosis; Top right: merged fluorescence video of PGCC-1 for demonstrating MEM; Lower left: merged fluorescence video of PGCC-2 for demonstrating RMEM; Lower right: Line chart showing the time interval of cell cycle phases of a diploid reference cell and its progeny cells. The time interval between each frame is 15 minutes. The frame rate for coding the uncompressed video file was 10 FPS. Bar equals 50 μm.

***SV-05***

This video demonstrates a representative decellularization process of a partly cellularized PGCC as a type 2 organoid under a 3D culture condition. Top left: merged images of LCK-GFP, gray; H2B-mCherry, magenta. Top right: LCK-GFP, gray. Lower left: phase-contrast, gray—lower right: H2B-mCherry, color-coded. The time interval between each frame is 15 minutes. The frame rate for coding the uncompressed video file was 10 FPS. Bar equals 25 μm.

***SV-06***

**SV06-SECTION 1**

This video demonstrates a representative process of forming a fecundity cell via amitotic mechanisms under a 3D culture condition. Left panels: merged images of LCK-GFP, gray; H2B-mCherry, magenta. Middle panels: H2B-mCherry, color-coded. Right column: LCK-GFP, color-coded. The time interval between each frame is 15 minutes. The frame rate for coding the uncompressed video file was 10 FPS. Bar equals 50 μm.

**SV06-SECTION 2**

This video demonstrates a representative entosis process between two type 2 organoids to form a fecundity structure under a 3D culture condition. Left panels: merged images of LCK-GFP, gray; H2B-mCherry, magenta. Middle panels: H2B-mCherry, color-coded. Right panels: LCK-GFP, gray. The time interval between each frame is 15 minutes. The frame rate for coding the uncompressed video file was 10 FPS. Bar equals 25 μm.

***SV-07***

This video demonstrates the different proliferation patterns that sustain organoid growth in type 1 and type 2 organoids. The 3D cultured organoids were transferred to a 2D culture condition for imaging. For both panels, the videos were generated from the color-coded time-lapse images of the H2B-mCherry channel. The left panel shows a poorly cellularized organoid, which contains abundant PGCCs. The right panel shows a well-cellularized organoid dominated by uniform diploid/near-diploid cells. The frame rate for coding the uncompressed video file was 10 FPS. The time interval between each frame was 15 minutes. Bar equals 100 μm.

**Supplemental Material and Methods**

**3D culture of organoids**

HGSC organoids were cultured in 30% (v/v) Matrigel matrix (354230, Corning) mixed with serum-free culture medium prepared in our laboratory with advanced DMEM/F12 reduced serum medium (12634-010, Gibco) supplemented with 50 ng/ml EGF (E9644-5, Sigma), 250 ng/ml R-SPONDIN1 (120-38-100UG, Peprotec), 0.5X B27 supplement (17504-044, Gibco), 0.5X N2 supplement (17502-048, Gibco), 1.25 mM N-acetylcysteine (A9165, Sigma), 50 nM A83-01 (SML0788, Sigma), 1X GlutaMAX (35050-061, Gibco), and 100 μg/ml Primocin (ant-pm-2, InvivoGen). The serum-free culture medium was adjusted to pH 7.2 using 0.1 M NaOH water solution, then filtered through 0.2-μm-pore Corning bottle-top vacuum filter systems for sterilization (CLS430769, Corning). The medium was aliquoted into 50-ml conical tubes and stored at -80°C. Before use, beta-mercaptoethanol was freshly added at a final concentration of 0.1 mM (21985-023, Gibco). The organoids were maintained at 37°C in a 5% CO_2_ humidified incubator. A modified medium was applied for organoids cultured during long-term imaging. This medium will be referred to as the advanced imaging medium. This advanced imaging medium was prepared based on FluoroBrite medium (12634-010, Gibco), with the rest supplements unchanged.

**Lentivirus packaging**

Lentiviral plasmids pBOB-LCK-GFP ([RRID: Addgene_118738](https://scicrunch.org/resolver/Addgene_118738)) (for cell membrane labeling), pLenti6-H2B-mCherry (RRID: Addgene_89766)(for nucleus/chromatin labeling), L304-EGFP-Tubulin-WT (RRID: Addgene_64060) (for cytoskeleton/spindle labeling), and pBOB-EF1-FastFUCCI-Puro ([RRID: Addgene_86849](https://scicrunch.org/resolver/Addgene_86849)) (cell cycle indicator) were purchased from Addgene as bacteria in agar stabs. The bacteria were subcloned and subjected to plasmid extraction.

For packing lentivirus particles, each above lentiviral vector was simultaneously transfected into HEK293TN cells ([RRID: CVCL_UL49](https://scicrunch.org/resolver/CVCL_UL49)) with two additional packing vectors: psPAX2 ([RRID: Addgene_12260](https://scicrunch.org/resolver/Addgene_12260)), which carries gag and pol, and pMD2.G ([RRID: Addgene_12259](https://scicrunch.org/resolver/Addgene_12259)), which carries VSV-G envelope. Standard transfection was performed using FuGENE 6 transfection reagent (E2691, Promega). The HEK293TN cells were cultured in 10-mm tissue culture dishes with antibiotic-free high-glucose DMEM (11965118, Gibco). At 48 hours after transfection, the supernatant laden with pseudoviral particles was harvested and filtered using 0.45-μm-pore syringe filters.

**Infection of organoids**

HGSC organoids were disassociated from the Matrigel matrix using Organoid Harvesting Solution (3700-100-01, Cultrex). The organoids were further dissociated into single-cell suspensions by incubation with 1X TrypLE Express (12604013, Gibco) at the final concentration. The single cells were centrifuged at 300 g for 5 minutes to obtain cell pellets, which were resuspended in a pre-warmed serum-free culture medium. For infection, approximately 3*10^5^ cells were seeded into a 24-well ultra-low attachment plate (Corning) with 1 ml of serum-free culture medium containing 8 μg/ml polybrene, then mixed with 1 ml of lentiviral supernatant.

**Flow cytometry and fluorescence-activated cell sorting**

The double-positive cell populations of the LCK-GFP/Histone H2B-mCherry-labeled and alpha tubulin-GFP/Histone H2B-mCherry-labeled organoids were purified using a MoFlo Astrios cell sorter. A 488-nm laser was used to excite GFP, and a 530-nm laser was used to excite mCherry. The purification of the cells labeled with the FUCCI system was carried out using a two-step method. In the first round of sorting, the cells solely expressing CDT-1 mKO2 (G1 marker) and GEMININ-mAG (G2/M marker) were separately collected. Then the two groups of isolated cells were cultured in 75-cm^2^ flasks until reaching 70% confluence. Then the two groups of cells were subjected to the second round of flow cytometric sorting. For the CDT-1 mKO2-positive cells sorted in the first round, the GEMININ-mAG solely positive cells were collected in the second-round sorting. Likewise, for the GEMININ-mAG solely positive cells sorted in the first round, the CDT-1 mKO2-positive cells were collected. Then the two groups of cells obtained from the second round of sorting were mixed, cultured, and subjected to further studies.

A BD FACSCalibur and a BD LSR II flow cytometer were applied to measure the fluorescence of the sorted cells for quality control.

**Fluorescence time-lapse imaging**

For imaging, the HGSC organoids were embedded in a 45% phenol red-free Matrigel matrix (356231, Corning) mixed with 55% advanced imaging medium on a glass-bottom culture plate (0.16-0.19 mm thick) (801004, NEST). For a six-well plate, 4 ml of advanced imaging medium was added to each well to support organoid growth during imaging.

A Lionheart (Biotek) multifunction imaging instrument was used to perform long-term time-lapse live-cell imaging. A two-channel gas controller was attached to the Lionheart to provide 5% CO_2_ inside the imaging chamber, and a 1450006 model insert stage was assembled for the instrument to maintain saturated humidity. Four functional modules were installed on the filter slides: 1) a laser autofocus cube (PN 1225010); 2) a DAPI filter cube assembly (LED model: 1225007, central wavelength 365 nm; filter cube model: 1225100, excitation 377/50 nm, emission 447/60 nm, dichroic mirror 409 nm); 3) a GFP filter cube assembly (LED model: 1225001, central wavelength 465 nm; filter cube model: 1225101, excitation 469/35 nm, emission 525/39 nm, dichroic mirror 497 nm); and 4) a Texas Red filter cube assembly (LED model: 1225002, central wavelength 590 nm; filter cube model: 1225102, excitation 586/15 nm, emission 647/57 nm, dichroic mirror 605 nm). The general imaging program was deployed in Gen5+ software as follows: 1) set the temperature to 37°C and reach stabilization; 2) perform laser autofocus; 3) camera start imaging with GFP filter cube, e.g., GFP-labeled alpha-tubulin or cell membrane (LCK-GFP); 3) 500-ms stabilization for the motor stage; 4) camera start imaging with Texas Red filter cube for H2B-mCherry; 5) move to the next beacon; 6) 500-ms stabilization; and 7) restart the imaging cycle from 1 to 4 and complete steps. For most imaging tasks, the time intervals were set to 15 minutes between each beacon. Depending on the experiments, Z-axis stacks comprising 3 to 5 slices were assigned to each fluorescence channel. The raw images generated by the Lionheart were further processed and analyzed with Gen5+ software, and a built-in 2D deconvolution module was applied. Fiji ImageJ software ([RRID: SCR_002285](https://scicrunch.org/resolver/SCR_002285)) (version 1.52p) software was used to complete quantitative measurements, video coding, and multiple-color rendering. The nuclear to cytoplasmic ratio was calculated by the formula R_N/C_=V_N_ /(V_C_ - V_N_), where R_N/C_ is the nuclear to cytoplasmic ratio, V_N_ is the volume of the nucleus, and V_C_ is the volume of the cytoplasm.

**Laser confocal and regular wide-field imaging**

An Andor Revolution XDi WD Spinning Disk confocal system was used to obtain high-resolution 3D images of live HGSC organoids. The system was equipped with an iXon Ultra 888 EMCCD camera and a Zyla 4.2 sCMOS camera. The raw images were acquired by using Andor iQ3 software. The greyscale raw images were further processed using Fiji software (Fiji is just ImageJ) (RRID: SCR_002285) (version 1.52p) for pseudo-color rendering, 3D reconstruction, and denoising. The third-party Image plugin PureDenoise (version 13/03/2014, Biomedical Imaging Group, Ecole Polytechnique Fédérale de Lausanne, Switzerland) was utilized with permission for noncommercial use.

Regular wide-field epifluorescence images were obtained with a Nikon TE2000U microscope equipped with a standard mercury lamp and a SPOT Flex camera. For immunofluorescence staining, the primary antibodies included monoclonal mouse anti-Vimentin (ab8978, Abcam)(dilution ratio: 1:200) and monoclonal rabbit anti-Sodium Potassium ATPase (ab8978, Abcam)(dilution ratio: 1:200). The secondary antibodies included goat anti-mouse Alexa Fluor™ 488 (A10680, Invitrogen)(dilution ratio: 1:200) and goat anti-rabbit Alexa Fluor™ 594 (A11072, Invitrogen)(dilution ratio: 1:200). The nuclei were counterstained by 1 μg/ml DAPI at a final concentration (D21490, Invitrogen) during the co-incubation with the secondary antibodies.

Phase contrast images of HGSC organoids were obtained with an Olympus IX71 inverted microscope. The color images of H&E–stained sections of patients' tumors and organoids were obtained with an Olympus BX41 microscope. Standard procedures were applied to prepare the paraffin slides and perform H&E-staining.
